# Stellate ganglion block ameliorated central post-stroke pain with comorbid anxiety and depression through inhibiting HIF-1α/NLRP3 signaling following thalamic hemorrhagic stroke

**DOI:** 10.1186/s12974-023-02765-2

**Published:** 2023-03-21

**Authors:** Zhong-Mou Shi, Jun-Jie Jing, Zheng-Jie Xue, Wen-Jun Chen, Yan-Bin Tang, Du-Juan Chen, Xin-Yi Qi, Li Huang, Yi-Qing Zou, Xiao-Zhi Wu, Fei Yang

**Affiliations:** 1 Department of Anesthesiology and Perioperative Medicine, Fuzong Clinical College (900th Hospital of the Joint Logistic Support Force), Fujian Medical University, Fuzhou, 350025 China; 2 Department of Anesthesiology and Perioperative Medicine, Dongfang Hospital, Xiamen University, Fuzhou, 350025 China; 3 Department of Neurosurgery, Fujian Children’s Hospital, College of Clinical Medicine for Obstetrics and Gynecology and Pediatrics, Fujian Medical University, Fuzhou, 350025 China; 4 Pain Research Institute, Fujian Medical University, Fuzhou, 350025 China

**Keywords:** Central post-stroke pain, Stellate ganglion block, Depression, Anxiety, Thalamus, Hypoxia-inducible factor 1α, NLRP3, Neuroinflammation

## Abstract

**Background:**

Central post-stroke pain (CPSP) is an intractable and disabling central neuropathic pain that severely affects patients’ lives, well-being, and socialization abilities. However, CPSP has been poorly studied mechanistically and its treatment remains challenging. Here, we used a rat model of CPSP induced by thalamic hemorrhage to investigate its underlying mechanisms and the effect of stellate ganglion block (SGB) on CPSP and emotional comorbidities.

**Methods:**

Thalamic hemorrhage was produced by injecting collagenase IV into the ventral-posterolateral nucleus (VPL) of the right thalamus. The up-and-down method with von Frey hairs was used to measure the mechanical allodynia. Behavioral tests were carried out to examine depressive and anxiety-like behaviors including the open field test (OFT), elevated plus maze test (EPMT), novelty-suppressed feeding test (NSFT), and forced swim test (FST). The peri-thalamic lesion tissues were collected for immunofluorescence, western blotting, and enzyme-linked immunosorbent assay (ELISA). Genetic knockdown of thalamic hypoxia-inducible factor-1α (HIF-1α) and NOD-like receptor thermal protein domain associated protein 3 (NLRP3) with microinjection of HIF-1α siRNA and NLRP3 siRNA into the VPL of thalamus were performed 3 days before collagenase injection into the same regions. Microinjection of lificiguat (YC-1) and MCC950 into the VPL of thalamus were administrated 30 min before the collagenase injection in order to inhibited HIF-1α and NLRP3 pharmacologically. Repetitive right SGB was performed daily for 5 days and laser speckle contrast imaging (LSCI) was conducted to examine cerebral blood flow.

**Results:**

Thalamic hemorrhage caused persistent mechanical allodynia and anxiety- and depression-like behaviors. Accompanying the persistent mechanical allodynia, the expression of HIF-1α and NLRP3, as well as the activities of microglia and astrocytes in the peri-thalamic lesion sites, were significantly increased. Genetic knockdown of thalamic HIF-1α and NLRP3 significantly attenuated mechanical allodynia and anxiety- and depression-like behaviors following thalamic hemorrhage. Further studies revealed that intra-thalamic injection of YC-1, or MCC950 significantly suppressed the activation of microglia and astrocytes, the release of pro-inflammatory cytokines, the upregulation of malondialdehyde (MDA), and the downregulation of superoxide dismutase (SOD), as well as mechanical allodynia and anxiety- and depression-like behaviors following thalamic hemorrhage. In addition, repetitive ipsilateral SGB significantly restored the upregulated HIF-1α/NLRP3 signaling and the hyperactivated microglia and astrocytes following thalamic hemorrhage. The enhanced expression of pro-inflammatory cytokines and the oxidative stress in the peri-thalamic lesion sites were also reversed by SGB. Moreover, LSCI showed that repetitive SGB significantly increased cerebral blood flow following thalamic hemorrhage. Most strikingly, SGB not only prevented, but also reversed the development of mechanical allodynia and anxiety- and depression-like behaviors induced by thalamic hemorrhage. However, pharmacological activation of thalamic HIF-1α and NLRP3 with specific agonists significantly eliminated the therapeutic effects of SGB on mechanical allodynia and anxiety- and depression-like behaviors following thalamic hemorrhage.

**Conclusion:**

This study demonstrated for the first time that SGB could improve CPSP with comorbid anxiety and depression by increasing cerebral blood flow and inhibiting HIF-1α/NLRP3 inflammatory signaling.

**Supplementary Information:**

The online version contains supplementary material available at 10.1186/s12974-023-02765-2.

## Introduction

Central post-stroke pain (CPSP), occurring after hemorrhagic or ischemic stroke concerning the somatosensory system, is categorized as central neuropathic pain syndrome. The prevalence rate of CPSP is reported at 8–46% following a hemorrhagic stroke due to the heterogeneity of affected brain areas, with a higher chance in patients who suffer a thalamic stroke [1, 2]. This intolerable and persistent pain condition severely affects the patient's quality of life and rehabilitation and imposes enormous medical and financial burdens. However, current pharmacological and non-pharmacological regimens for CPSP are far from satisfactory and often result in undesirable side effects [3–5]. The pathophysiology of CPSP remains largely unknown, which hinders the development of novel therapeutic strategies. More seriously, CPSP is frequently comorbid with psychiatric disorders, in particular anxiety and depression, which exacerbate the duration and severity of pain and drive a vicious cycle between pain and negative emotion, ultimately making CPSP more resistant to therapy [6–8]. Thus, there is an urgent need to understand the mechanisms underlying CPSP with comorbid anxiety and depression and to explore a new and effective treatment for these conditions.

Accumulating evidence indicates that inflammatory responses involving the immunologically active glial cells and the pro-inflammatory cytokines and chemokines play an essential role in ischemic or hemorrhagic stroke. It has been demonstrated that the maladaptive neuroinflammation following stroke contributes to the disease process in CPSP. Systemic depletion of microglia with PLX3397 effectively prevents mechanical allodynia caused by thalamic hemorrhagic stroke [9]. Inhibition of local inflammatory cytokines (IL-1β, TNF-α) and chemokines (CXCL12/CXCR4) signaling as well as glial cell activation in the damaged thalamus also markedly ameliorated CPSP symptoms transiently [10–14]. Notably, microglia activation has also been implicated in thalamic hemorrhage-induced depression [15], suggesting that targeting microglia-mediated inflammatory cascades may be rational for treating CPSP and emotional comorbidity. Our previous study revealed that hypoxia-inducible factor-1 alpha (HIF-1α), an oxygen-dependent transcriptional activator, was the initiator of neuroinflammation following thalamic hemorrhagic stroke and was involved in the genesis of CPSP by boosting glial cell activation and driving the expression of pro-inflammatory cytokines [14]. Recent studies showed that the NOD-like receptor thermal protein domain associated protein 3 (NLRP3) inflammasome, an essential downstream target of HIF-1α after stroke, was extremely upregulated in the damaged thalamus of the CPSP model, and the NLRP3 inflammasome inhibitor was able to attenuate CPSP syndrome [16–19]. Moreover, inhibiting HIF-1α/NLRP3 pathway could improve inflammation-induced depressive-like behaviors [20]. Thus, exploiting a novel and effective approach targeting the HIF-1α/NLRP3 signaling-mediated neuroinflammation may open a new avenue for treating CPSP and emotional comorbidity.

Stellate ganglion block (SGB) is a commonly used and effective method for temporarily blocking the cervical sympathetic trunk. A large body of data suggests that SGB significantly improves the prognosis of cerebrovascular events by alleviating cerebral vascular spasm, increasing brain oxygen supply, reducing the inflammatory response, and decreasing oxidative stress [21–24]. Recently, SGB has emerged as a novel treatment for various pathological pain, such as complex regional pain syndrome, postoperative pain, and orofacial pain [25–27]. For CPSP, case studies showed that a single SGB treatment considerably alleviated somatic pain and decreased the usage of analgesic medicines in CPSP patients and that the analgesic effects of a single SGB therapy lasted at least one month [28, 29]. However, the underlying analgesic mechanisms of SGB on CPSP remain unclear. In addition, experimental and clinical studies have shown that SGB exhibits a significant protective effect against anxiety and depression-like behaviors [30–33]. In the light of these findings, we are therefore interested to know whether SGB could improve emotional comorbidities associated with CPSP, and if so, what the cellular and molecular mechanisms are.

In this study, we provide preclinical evidence that addresses the CPSP and its anxious and depressive consequences using a reproducibly established thalamic hemorrhagic stroke model and investigate the role of SGB in inhibiting HIF-1α/NLRP3 signaling to improve CPSP with comorbid anxiety and depression.

## Materials and methods

### Animals

A total of 260 male Sprague-Dawley rats weighing 200–250 g were used for this study. Among the 260 rats, 13 were excluded from the study due to death during the experiment. All rats were provided by the Laboratory Animal Center of 900th Hospital. Rats were housed five per cage under controlled temperature (23 ± 1 °C) and humidity (50 ± 5%) with a 12 h light/12 h dark cycle and free access to food and water. All rats were acclimated in the test room for 5–7 days before behavioral experiments and all behavioral tests were conducted between 9:00 and 16:00 by using blind methods. Rats were randomly assigned throughout the whole trial. This study was conducted following the Animal Care and Use Committee of 900th Hospital (Authorization No.: 2021-006) and the National Institutes of Health guide for the care and use of laboratory animals (NIH Publications No. 8023, revised 1978). The number of rats used was minimized, as was their suffering.

### CPSP surgery

Surgery was performed as previously described [14, 34, 35]. Rats were anesthetized with 3% isoflurane and placed on a stereotaxic apparatus (RWD Life Technology, Shenzhen, China), and maintained with 1.5–2.0% isoflurane via nose cone at a speed of 0.5 L/min. After cutting the scalp, swab the periosteum with a sterile dry cotton ball to separate the periosteum to expose the skull. Craniotomies were performed with a pneumatic drill to cause minimal damage to cortical tissue. Collagenase type IV (0.025 U/0.25 μl, Sigma-Aldrich China, Shanghai) or saline (0.25 μl) were microinjected into the ventral-posterolateral nucleus (VPL) of the right thalamus using a 1-µl syringe and 33-gauge needle. The stereotaxic coordinates of the injections were 3.48 mm anterior–posterior to the bregma, 3.6 mm lateral to the midline, and 6.2 mm ventral to the brain surface to target the VPL. After each injection, the need was left for approximately 5 min to prevent the agent from spreading to the surface of the brain. The needle was then slowly withdrawn, and the skin was sutured with 4.0 sutures. During the surgery, the rats were placed on a thermal blanket maintained at 37 °C to prevent hypothermia. After the surgery, all rats were allowed to recover in individual cages for 7 days with free access to food and water. Naïve rats were fed under the same conditions in a parallel manner.

### Intra-thalamic drug injections

Rats were intra-thalamic injected with lificiguat (YC-1, Selleck, 0.2 mM) or MCC950 (Selleck, 0.2 mM) in a total volume of 1 μl over 10 min and which was performed 30 min before collagenase injection. The doses used in this study were based on previous studies [14, 36]. YC-1, a well-established HIF-1α inhibitor, was used to confirm the regulatory effects of HIF-1α on the development of CPSP. MCC950, which is an NLRP3 selective inflammasome-specific inhibitor, was used to evaluate the roles of NLRP3 in the development of CPSP. Both YC-1 and MCC950 were dissolved in 1% dimethyl sulfoxide (DMSO). The control group received an equal volume intra-thalamic injection of the DMSO vehicle. To confirm that thalamic HIF-1α/ NLRP3 signaling mediated the therapeutic effect of SGB, we pharmacologically activated thalamic HIF-1α and NLRP3 by intra-thalamic injection of nigericin (Selleck, 0.2 mM, 1 μl) and DMOG (Selleck, 0.2 mM, 1 μl) at 9, 11, and 13 days in rats given repetitive SGB after intra-thalamic collagenase injection. The intra-thalamic injection methods were identical to the above-mentioned and the injection locations were examined histologically.

### siRNA preparation and microinjection

The sequence of HIF-1α siRNA (sense: 5′-GGAAACGAGUGAAAGGAUATT-3′; anti-sense: 5′-UAUCCUUUCACUCGUUUCCAA-3′) and NLRP3 siRNA (sense, 5′ -GCAUGCACGUCUAAUCUCUTT-3′; anti-sense, 5’ -AGAGAUUAGACGUGCAUGCAT-3′) and negative control scrambled siRNA (sense, 5′-UUCUCCGAACGAGUCACGUTT-3′; anti-sense, 5′-ACGUGACUCGUUCGGAGAATT-3′) was designed and chemically synthesized by SunYa Biotechnology (Fuzhou, China). The RNase-free water and in vivo transfection reagent was used to dissolve siRNA reaching the final concentration of 2 μmol/L. 10 μL of the diluted siRNA solution was microinjected into the ventral-posterolateral nucleus (VPL) of the right thalamus in the same manner as the Collagenase type IV microinjection described above.

### Stellate ganglion block

Rats were anesthetized by inhalation of 3% isoflurane and then placed prone on an operating table. The cartilaginous process of the spinous process of the seventh cervical vertebra was palpated, and a 1-ml syringe needle was inserted forward along the right sagittal position of the seventh cervical vertebra. When the needle tip lost contact with the vertebral body, it was slightly retracted about 0.5 mm. After confirming that no blood or cerebrospinal fluid is seen in the retracted syringe, 0.2 ml of 0.25% ropivacaine was injected, while the control group received an equal volume of saline injection. After awakening from anesthesia, the typical Horner syndromes such as sagging eyelids, narrowed eye fissures, and constricted pupils on the block side in SGB rats were the signs of a successful block. Otherwise, we judged the SGB was unsuccessful, and these rats were excluded from the study.

### Mechanical pain sensitivity testing

To evaluate mechanical allodynia, mechanical stimuli were applied using a series of von Frey monofilaments with varying bending forces. The rats were placed on a metal mesh floor covered by a 20 × 20 × 20 cm transparent plastic box and acclimatized for 30 min. Von Frey filaments were applied from underneath the metal mesh floor to the plantar surface of the hind paws vertically. The mechanical stimulus began with the smallest bending force and progressively increased in intensity. Each von Frey filament was applied 10 times (once every several seconds) to elicit the withdrawal response. Paw withdrawal mechanical threshold (PWMT, g) was defined as the lowest von Frey filament bending force value at which paw withdrawal occurred at a frequency of 50%. The PWMT of bilateral hindpaws was measured.

### Open field test (OFT)

The rats were acclimated to the test room for at least 1 h before the OFT. During the experiment, the test room was kept at a constant temperature and humidity (24 °C and 55% relative humidity) with dim lighting and no noise. The bottom of the 100 × 100 × 40 cm black open field box was divided into 16 grids, with the center 4 grids being the central area and the other 12 grids being the surrounding regions. The rats were placed gently in the center area of the open field box and allowed to move freely for 5 min. The time spent and the traveled distance in the center area as well as the total traveled distance were recorded and analyzed by video tracking software (SMART v.3.0 software, RWD Life Science). The less time and distance in the central area indicated the higher the anxiety level. After each experiment, clean the box carefully with 75% ethanol to remove rat excrement and odor. The following experiment will be conducted once the ethanol has evaporated.

### Elevated plus maze test (EPMT)

The elevated plus-maze apparatus consists of 4 arms (10 × 50 cm) and a central platform (10 × 10 cm) elevated 50 cm above the floor. Two closed arms were enclosed with 30-cm-high walls crossing with two open arms (with no walls). The rats were placed in the central area facing an open arm and were allowed to freely explore the maze for 5 min. The time spent and the traveled distance in the open arm as well as the total traveled distance were recorded and analyzed by video tracking software (SMART v.3.0 software, RWD Life Science). The less time and distance in the open arm indicated the higher the anxiety level. After each experiment, clean the box carefully with 75% ethanol to remove rat excrement and odor. The following experiment will be conducted once the ethanol has evaporated.

### Novelty-suppressed feeding test (NSFT)

The was performed as described previously [37]. Briefly, 24 h before testing, the rats were food-deprived, and only water was available. During testing, each rat was placed in one corner of a plastic box (100 × 100 × 40 cm) with wooden bedding covered floor and allowed to explore for a maximum of 10 min. Three food pellets were placed on a circular filter paper in the center of the arena. The rats were placed in one corner of the box and the time taken to bite food, not simply sniff or touch the pellet was recorded as feeding latency. If a rat did not bite food within 10 min, the feeding latency was recorded as 10 min.

### Forced swim test (FST)

The rats were placed in a plastic cylinder (30 cm diameter, 50 cm height) containing 30 cm of water at a temperature of 25 ± 1 °C. The activities of rats during a 5-min test were recorded by the video system, and the accumulated immobility time was counted. Immobility was defined as the rats floating with no active movements other than those necessary to maintain the head and nose above the water. The duration of immobility was strongly linked with the severity of depression in rats. The rats were dried with a towel immediately and returned to their home cages after the test.

### Western blotting

Ipsilateral thalamus tissue around the hemorrhagic lesion sites was dissected from the brain of rats and lysed using RIPA lysis buffer containing protease and phosphatase inhibitors, and centrifuged at 12,000 rpm for 10 min. The total protein concentration of the lysate was measured using a BCA protein assay kit (Solarbio, Beijing, China), and heated at 100 °C for 10 min. Equal amounts of proteins were resolved by SDS-PAGE and then transferred to PVDF membranes (Millipore, Molsheim, France). Membranes were blocked with 5% skim milk (Solarbio, Beijing, China) at room temperature for 1 h and then incubated with primary antibodies overnight at 4 °C. Primary antibodies included rabbit anti-HIF-1α (1:1000, Bioswamp, Wuhan, China), rabbit anti-Caspase 1 (1:1000, Bioswamp, Wuhan, China), rabbit anti-NLRP3 (1:1000, Bioswamp, Wuhan, China), and rabbit anti-Iba-1 (1:1000, Bioswamp, Wuhan, China), rabbit anti-GFAP (1:1000, Bioswamp, Wuhan, China). On the following day, membranes were washed three times with PBST and then incubated with HRP-conjugated secondary antibody (Goat anti-Rabbit IgG, 1:20,000, Bioswamp, Wuhan, China) for 1 h at room temperature. Membranes were visualized with a chemiluminescent solution (Millipore, Molsheim, France) and detected in an automatic chemiluminescence analyzer (Tanon, Shanghai, China) with TANON GIS software (Tanon, Shanghai, China). The scanned images were quantified using ImageJ software. Specific bands for each protein were normalized to its respective β-actin control.

### Enzyme-linked immunosorbent assay (ELISA)

Ipsilateral thalamus tissue around the hemorrhagic lesion sites was dissected from the brain of rats and lysed using RIPA lysis buffer containing protease and phosphatase inhibitors, and centrifuged at 12,000 rpm for 10 min. After quantitative measurement of the total protein concentrations with a BCA protein assay kit (Solarbio, Beijing, China), the homogenized thalamus tissue was assayed for HIF-1α, TNF-α, IL-1β, IL-6, IL-33, CCL-12, MDA and SOD by ELISA kits (Bioswamp, Wuhan, China). Each protein of interest was expressed as picograms or nanogram per milligram of total proteins and SOD-specific activity is represented as U/mg of protein.

### Immunofluorescence

Rats were anesthetized with pentobarbital sodium (50 mg/ kg, i.p.) and transcardially perfused with saline followed by 4% paraformaldehyde. Brain tissues were quickly removed, and postfixed overnight in 4% paraformaldehyde at 4 °C. After tissue dehydration/rehydration, brains were paraffin-embedded. The thalamus region was sectioned into 10-μm-thick slices using a microtome, and the sections were deparaffined in xylene and hydrated through a series of graded ethanol. Antigens were activated by microwave heating in EDTA antigen repair buffer (pH = 8.0) for 10–30 min. After washing in PBS, sections were incubated with normal goat serum for 1 h at room temperature, followed by overnight incubation at 4 °C in a humid chamber with primary antibodies, rabbit anti-Iba-1 (1:200, Bioswamp, Wuhan, China) or rabbit anti-GFAP (1:200, Bioswamp, Wuhan, China). The sections were then washed and incubated for 1 h at room temperature with an anti-rabbit secondary antibody (1:300, GB21303, Servicebio, Wuhan, China). The images were examined and captured under a fluorescent microscope (Nikon, Japan), and the quantification of cell count was completed with ImageJ software.

### Morphological analysis of microglia

As previously described, the morphology of microglia was examined by the skeleton and fractal analysis using the ImageJ software [38]. Briefly, the immunofluorescence images were first converted to binary images, followed by skeletonization using Image J software. The Analyze Skeleton (2D/3D) plugin was then used in all skeletonized images to identify elements of the microglial cytoskeleton, and the number of endpoints and the length of processes were examined automatically.

### Laser speckle contrast imaging (LSCI)

The rats were anesthetized with 3% isoflurane and placed on a stereotaxic apparatus (RWD Life Technology, Shenzhen, China), and maintained with 1.5–2.0% isoflurane via nose cone at a speed of 0.5 L/min. After making a 15-mm midline skin incision, a high-speed skull drill was used to thin the skull (10 mm × 10 mm) under a microscope until the blood veins were visible. The drill was cooled repeatedly with saline to avoid heat damage to brain tissue. The LSCI system (Wuhan SIM Optoelectronics Technology Co., Wuhan, China) includes an Olympus ZS61 microscope, a continuous wavelength laser diode (λ = 785 nm), and a charge-coupled device camera. The thinned skulls were illuminated vertically by the continuous wavelength (λ = 785 nm) laser source, and the images reflected by the biological tissue were captured by an in-system CCD imaging system. A random interference pattern is created due to the mutual interference of scattered light through different optical paths, and a pseudo-color image is generated by computer processing automatically. The configuration of blood vessels and the direction of blood flow on the surface of brain tissue were examined under a microscope. The blood flow index (BFI) reflected by the gray average value of the cortical blood flow image was examined and calculated by ImageJ software. All the rats were kept warm during the test and then housed in a single cage after awakening with access to food and water.

### Statistical analysis

Data are expressed as mean ± SEM. All data were statistically analyzed using SPSS 22.0 and GraphPad Prism 8.0. The sample sizes were based on our previous knowledge and experience with this design. The normality test was performed by the Shapiro–Wilk test. The homogeneity of variance test was performed by Levene’s test. Data that met these two conditions were analyzed by one-way, two-way, or two-way RM ANOVA followed by Tukey’s post hoc test. Data with unequal variance were compared by Welch ANOVA followed by Tamhane’s T2 post hoc test. A level of *p* < 0.05 was considered statistically significant.

## Result

### Thalamic hemorrhagic stroke induced mechanical allodynia and anxiety- and depression-like behaviors in rats

**Fig. 1:**
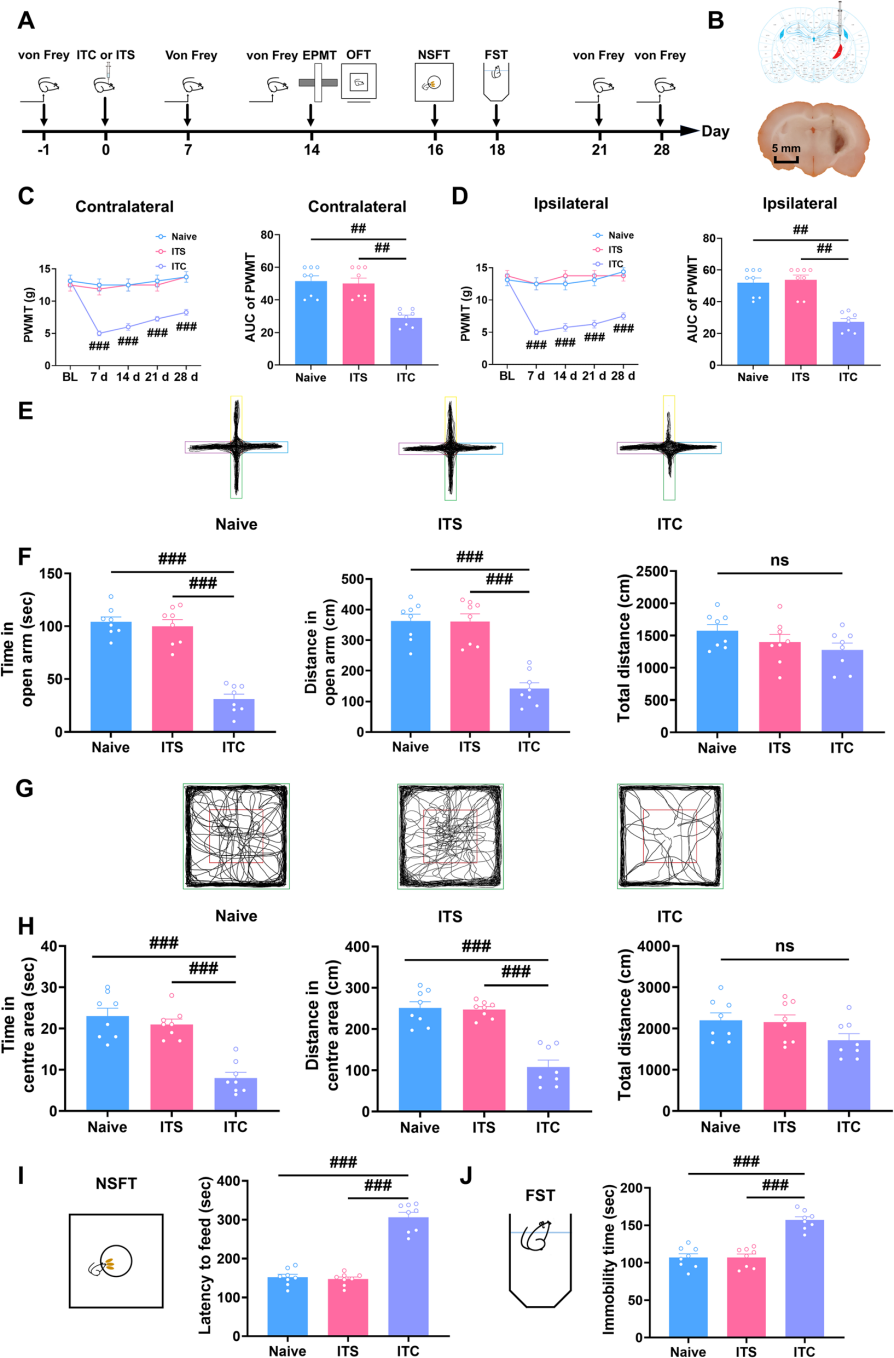
ITC rats exhibited mechanical allodynia and anxiodepressive-like behaviors. **A** Experimental timeline for surgical procedure and behavior tests. **B** Schematic diagram showing the injection site of ITC (top) and representative photomicrograph of brain slice showing the hemorrhagic lesion location following ITC (bottom). Scale bar = 1 mm. **C** The PWMT of the contralateral hindpaw was significantly decreased 7 days after ITC and persisted throughout the testing period, as did the area under curve of the contralateral hindpaw PWMT (*n* = 8, PWMT: group, *F*_2, 21_ = 15.53, day, *F*_4, 84_ = 25.63, group × day, *F*_8, 84_ = 16.45, ^###^*p* < 0.001 vs Naive; AUC: *F*_2, 21_ = 27.71, ^##^*p* < 0.01). **D** The PWMT of the ipsilateral hindpaw was significantly decreased 7 days after ITC and persisted throughout the testing period, as did the area under curve of the ipsilateral hindpaw PWMT (*n* = 8, PWMT: group, *F*_2, 21_ = 22.95, day, *F*_4, 84_ = 29.24, group × day, *F*_4, 84_ = 19.49, ^###^*p* < 0.001 vs Naive; AUC: *F*_2, 21_ = 19.18, ^##^*p* < 0.01). **E** Representative track plot in the EPMT. **F** ITC decreased the time spent and traveled distance in the open arms, but had no effect on the total traveled distance in EPMT (*n* = 8, time in open arm: *F*_2, 21_ = 61.14, ^###^*p* < 0.001; distance in open arm: *F*_2, 21_ = 32.31, ^###^*p* < 0.001; total distance: ns, no significance). **G** Representative track plot in the OFT. **H** ITC decreased the time spent and traveled distance in central area, but had no effect on the total traveled distance in the OFT (*n* = 8, time in central area: *F*_2, 21_ = 28.00, ^###^*p* < 0.001; distance in central area: *F*_2, 21_ = 35.95, ^###^*p* < 0.001; total distance: ns, no significance). **I** ITC increased the latency to feed in the NSFT (*n* = 8, *F*_2, 21_ = 38.23, ^###^*p* < 0.001). **J** ITC increased the immobility time in the FST (*n* = 8, *F*_2, 21_ = 97.32, ^###^*p* < 0.001). Data are expressed as mean ± SEM, one-way ANOVA followed by the Tukey test

The thalamic hemorrhagic stroke model was established by injecting collagenase into the right thalamic ventral-posterolateral nucleus to mimic clinical CPSP. Consistent with our previous studies [14, 34, 35], thalamic hemorrhagic stroke induced by intra-thalamic collagenase injection (ITC) developed bilateral mechanical allodynia within 7 days and persisted at least for 28 days (Fig. 1A–D). During the whole observation period, intra-thalamic saline injection (ITS) did not affect bilateral paw withdrawal mechanical threshold compared to the naïve group (Fig. 1A–D).

Neuropathic pain is frequently accompanied by psychiatric disorders, such as anxiety and depression [39–41]. Thus, on day 14 after intra-thalamic collagenase injection, when the mechanical allodynia is steady, we conducted the elevated plus maze test and open field test to evaluate anxiety-like behaviors (Fig. 1A). In the elevated plus maze test, ITC significantly reduced the time and traveled distance in open arms with no effect on the total traveled distance compared with naïve group. In accordance with the elevated plus maze test result, ITC markedly reduced the time and traveled distance in the central area in the open field test, but had no effect on the total traveled distance compared with the naïve group (Fig. 1E–H). There was no significant difference between the naive group and the ITS group in the elevated plus maze test and open field test (Fig. 1E–H). Next, we performed the novelty-suppressed feeding test and forced swim test 16 and 18 days after intra-thalamic injection to evaluate depressive-like behaviors, respectively (Fig. 1A). In the novelty-suppressed feeding test, ITC significantly increased the latency to feed, while ITS had no effect compared with the naïve group (Fig. 1I). In parallel, ITC significantly increased the immobility time, while ITS had no effect compared with naïve group in the forced swim test (Fig. 1J). These results suggest that ITC-induced CPSP rats exhibited anxiodepressive-like behaviors.

### ITC activated microglia and astrocytes and upregulated HIF-1α and NLRP3 in the peri-thalamic lesion sites

**Fig. 2:**
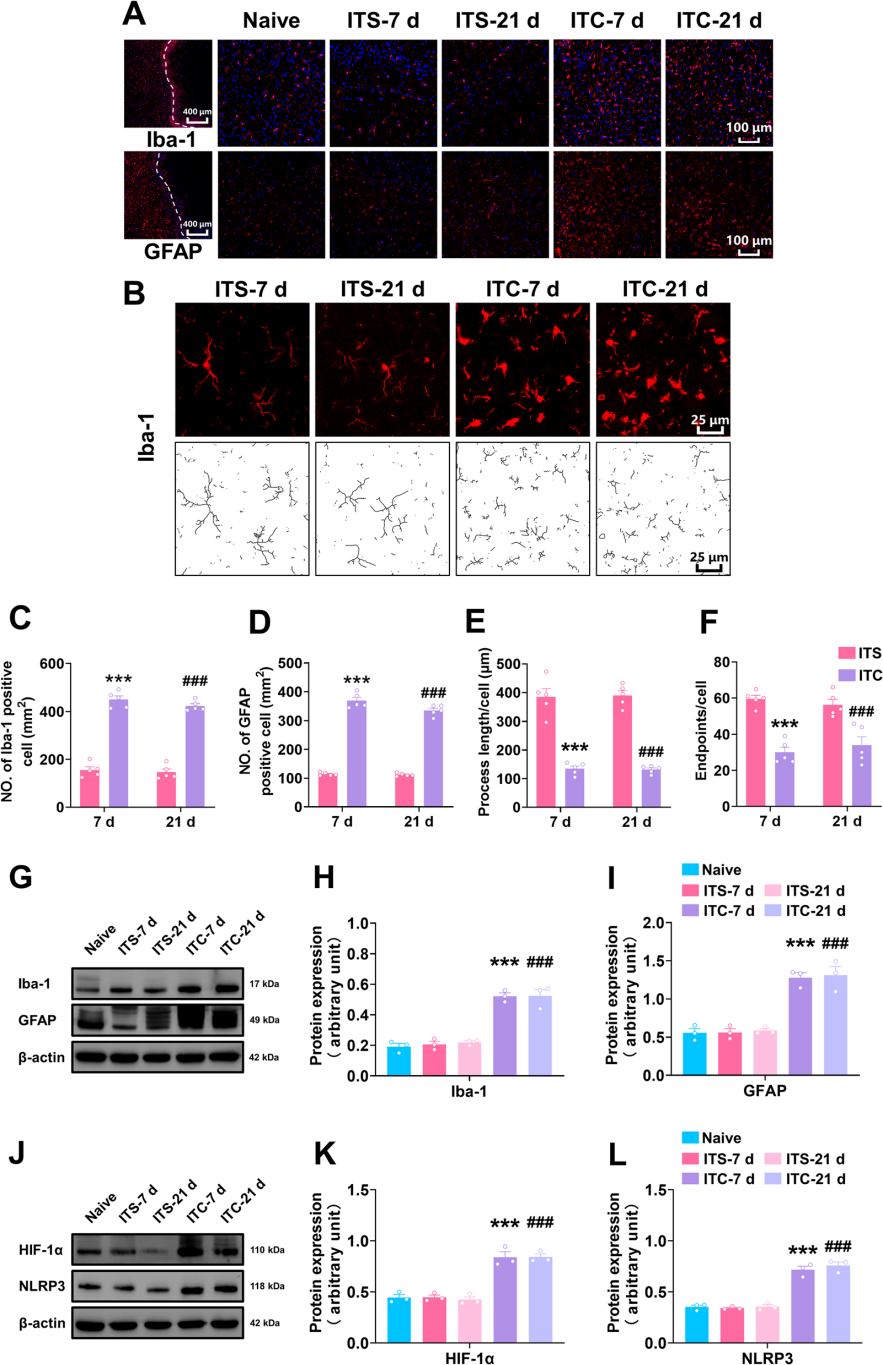
ITC activated microglia and astrocytes and upregulated HIF-1α and NLRP3 in the peri-thalamic lesion sites of rats. **A** Representative immunofluorescence images showing the time course expressions of GFAP and Iba-1, in the peri-thalamic lesion sites. Scale bar = 100 μm. **B** Representative magnified images of microglia (top) and the corresponding black-and-white, skeletonized images (bottom) in the peri-thalamic lesion sites. Scale bar = 25 μm. **C, D** Quantification of cell number showed ITC increased Iba-1 (**C**) and GFAP (**D**) positive cells in in the peri-thalamic lesion sites at 7 and 21 d *n* = 5, Iba-1: *F*_3, 16_ = 167.2, ***p < 0.001 vs ITS-7 d, ^###^*p* < 0.001 vs ITS-21 d; *n* = 5, GFAP: *F*_3, 16_ = 243.1, ***p < 0.001 vs ITS-7 d, ^###^*p* < 0.001 vs ITS-21 d). **E** Quantification of process length showed ITC reduced the process length of microglia at 7 and 21 d (*n* = 5, *F*_3, 16_ = 20.71, ***p < 0.001 vs ITS-7 d, ^###^*p* < 0.001 vs ITS-21 d). **F** Quantification of endpoint showed ITC decreased the endpoints in microglia at 7 and 21 d (*n* = 5, *F*_3, 16_ = 22.08, ***p < 0.01 vs ITS-7 d, ^###^*p* < 0.001 vs ITS-21 d). **G** Representative western blots of Iba-1 and GFAP expression in total proteins of the peri-thalamic lesion sites in naïve, ITS, and ITC rats.** H** Quantitative summary result showed ITC increased Iba-1 expression at 7 and 21 d, while ITS had no effect (*n* = 3, *F*_4, 10_ = 46.93, ***p < 0.001 vs ITS-7 d, ^###^*p* < 0.01 vs ITS-21 d). **I** Quantitative summary result showed ITC increased GFAP expression at 7 and 21d, while ITS had no effect (*n* = 3, *F*_4, 10_ = 33.41, ***p < 0.001 vs ITS- 7d, ^###^*p* < 0.001 vs ITS-21 d). **J** Representative western blots of HIF-1α and NLRP3 expression in total proteins of the peri-thalamic lesion sites in naïve, ITS, and ITC rats. **K** Quantitative summary result showed ITC increased HIF-1α expression at 7 and 21 d, while ITS had no effect (*n* = 3, *F*_4, 10_ = 91.24, ***p < 0.001 vs ITS-7 d, ^###^*p* < 0.001 vs ITS-21 d).** L** Quantitative summary result showed ITC increased NLRP3 expression at 7 and 21 d, while ITS had no effect (*n* = 3, *F*_4, 10_ = 67.22, ***p < 0.001 vs ITS-7 d, ^###^*p* < 0.001 vs ITS-21 d). Data are expressed as mean ± SEM, one-way ANOVA followed by Tukey test

To investigate the activation of microglia and astrocyte in the peri-thalamic lesion site induced by ITC, we performed immunofluorescent staining for glial fibrillary acidic protein (GFAP, an astrocyte marker) and ionized calcium binding adapter molecule 1Iba-1(Iba-1, a microglia marker) 7 and 21 days after intra-thalamic injection. As shown in Fig. 2A–D, ITC substantially enhanced the number of Iba-1 and GFAP-positive cells compared to the ITS group at 7 and 21 days. We further examine the morphological characterization of Iba-1-labeled cells in ITS and ITC group (Fig. 2B). At 7 and 21 days, the endpoints and process lengths of Iba-1-labeled cells were dramatically reduced in the ITC group relative to the ITS group (Fig. 2E and F). In conformity with the immunofluorescent results, western blotting showed the expression level of Iba-1 and GFAP in the peri-thalamic lesion site were highly increased in the ITC group at 7 and 21 days compared to the naïve group, whereas ITS had no influence on the expression level of Iba-1 and GFAP (Fig. 2G–I). These results suggest that microglia and astrocytes were significantly activated in the peri-thalamic lesion site in the CPSP rats. Our previous studies have demonstrated that HIF-1α is involved in the development of CPSP [14]. Here, we examined the expression of HIF-1α and NLRP3 by the western blotting approach. Compared to the naïve group, ITS did not affect the expression level of HIF-1α and NLRP3 at 7 and 21 days, whereas HIF-1α and NLRP3 were extremely upregulated in the peri-thalamic lesion site in the ITC group (Fig. 2J–L).

### Genetic knockdown or pharmacological inhibition of HIF-1α and NLRP3 prevented mechanical allodynia and anxiety- and depression-like behaviors in CPSP rats

**Fig. 3:**
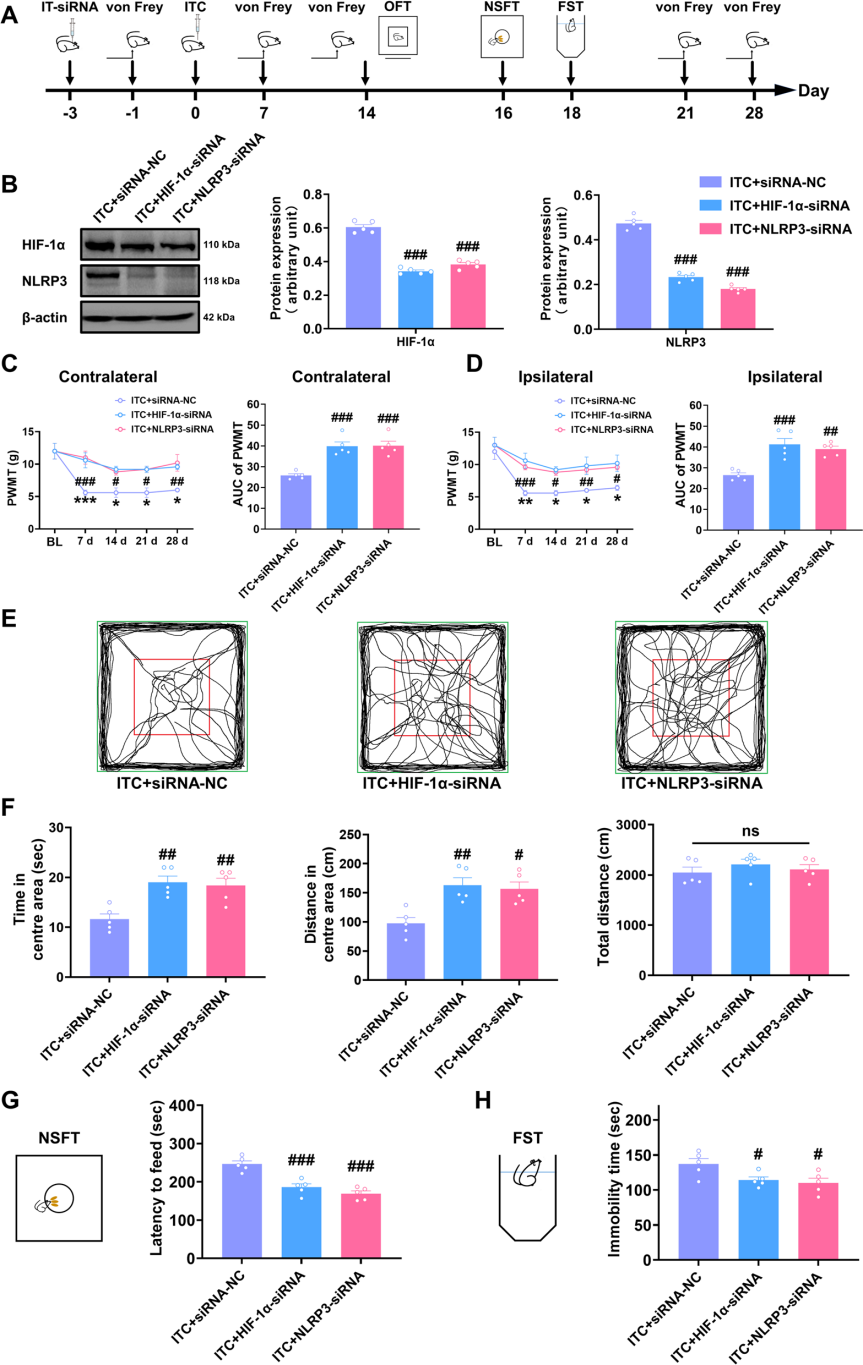
Intra-thalamic injection of HIF-1α siRNA or NLRP3 siRNA significantly prevented ITC-induced mechanical allodynia and anxiodepressive-like behaviors in rats. **A** The experimental timeline of surgical procedure and behavior tests. **B** Both HIF-1α siRNA and NLRP3 siRNA silenced the expression of HIF-1α and NLRP3 in total proteins of the peri-thalamic lesion sites in ITC rats. (*n* = 5, HIF-1α: *F*_2, 12_ = 143.9, ^###^*p* < 0.001 vs ITC + siRNA-NC; NLRP3: *F*_2, 12_ = 248.2, ^###^*p* < 0.001 vs ITC + siRNA-NC). **C** Temporal changes of PWMT in contralateral hindpaw after thalamic hemorrhagic stroke and the area under curve of the contralateral hindpaw PWMT (*n* = 5, PWMT: group, *F*_2, 12_ = 13.49, day, *F*_4, 48_ = 14.54, group × day, *F*_8, 48_ = 2.182, ^#^*p* < 0.05, ^##^*p* < 0.01, ^###^*p* < 0.001 ITC + siRNA-NC vs ITC + HIF-1α-siRNA, *p < 0.05, ***p < 0.001, ITC + siRNA-NC vs ITC + NLRP3-siRNA; AUC: *F*_2, 12_ = 20.89, ^###^*p* < 0.001). **D** Temporal changes of PWMT in ipsilateral hindpaw after thalamic hemorrhagic stroke and the area under curve of the ipsilateral hindpaw PWMT (*n* = 5, PWMT: group, *F*_2, 12_ = 11.54, day, *F*_4, 48_ = 21.28, group × day, *F*_12, 48_ = 2.796, ^#^*p* < 0.05, ^##^*p* < 0.01, ^###^*p* < 0.001, ITC + siRNA-NC vs ITC + HIF-1α-siRNA, *p < 0.05, **p < 0.01, ITC + siRNA-NC vs ITC + NL; RP3-siRNA; AUC: *F*_2, 12_ = 16.86, ^##^*p* < 0.01, ^###^*p* < 0.001). **E** Representative track plot in the OFT. **F** HIF-1α siRNA and NLRP3 siRNA increased the time spent and traveled distance traveled in central area, and but had no effect on the total traveled distance in the OFT (*n* = 5, time in central area: *F*_2, 12_ = 10.51, ^##^*p* < 0.01; distance in central area: *F*_2, 21_ = 9.466, ^#^*p* < 0.05; total distance: ns, no significance). **G** HIF-1α siRNA and NLRP3 siRNA decreased the latency to feed in the NSFT (*n* = 5, *F*_2, 12_ = 24.43, ^###^*p* < 0.001). **H** HIF-1α siRNA and NLRP3 siRNA decreased the immobility time in the FST (*n* = 5, *F*_2, 12_ = 5.035, ^#^*p* < 0.05). Data are expressed as mean ± SEM, one-way ANOVA followed by Tukey test

To confirm the involvement of HIF-1α and NLRP3 in CPSP and associated anxiety and depression, we knocked down thalamic HIF-1α and NLRP3 through microinjection of HIF-1α siRNA or NLRP3 siRNA into the VPL of thalamus 3 days before microinjection of collagenase into the same regions (Fig. 3A). As expected, the amount of HIF-1α and NLRP3 protein in the thalamus of ITC rats was decreased by pre-microinjection with HIF-1α siRNA or NLRP3 siRNA (Fig. 3B). Compared with the scrambled siRNA microinjection, pre-microinjection with HIF-1α siRNA or NLRP3 siRNA significantly prevented the bilateral mechanical allodynia at 7 to 28 days post-collagenase injection (Fig. 3C and D). In the open field test, pre-microinjection with HIF-1α siRNA or NLRP3 siRNA significantly increased the time and traveled distance in the central area compared to the control group, but had no effect on the total distance (Fig. 3E and F). Compared with the scrambled siRNA microinjection, pre-microinjection with HIF-1α siRNA or NLRP3 siRNA also reduced the feeding latency in novelty-suppressed feeding test (Fig. 3G) and the immobility in forced swim test (Fig. 3H).

**Fig. 4:**
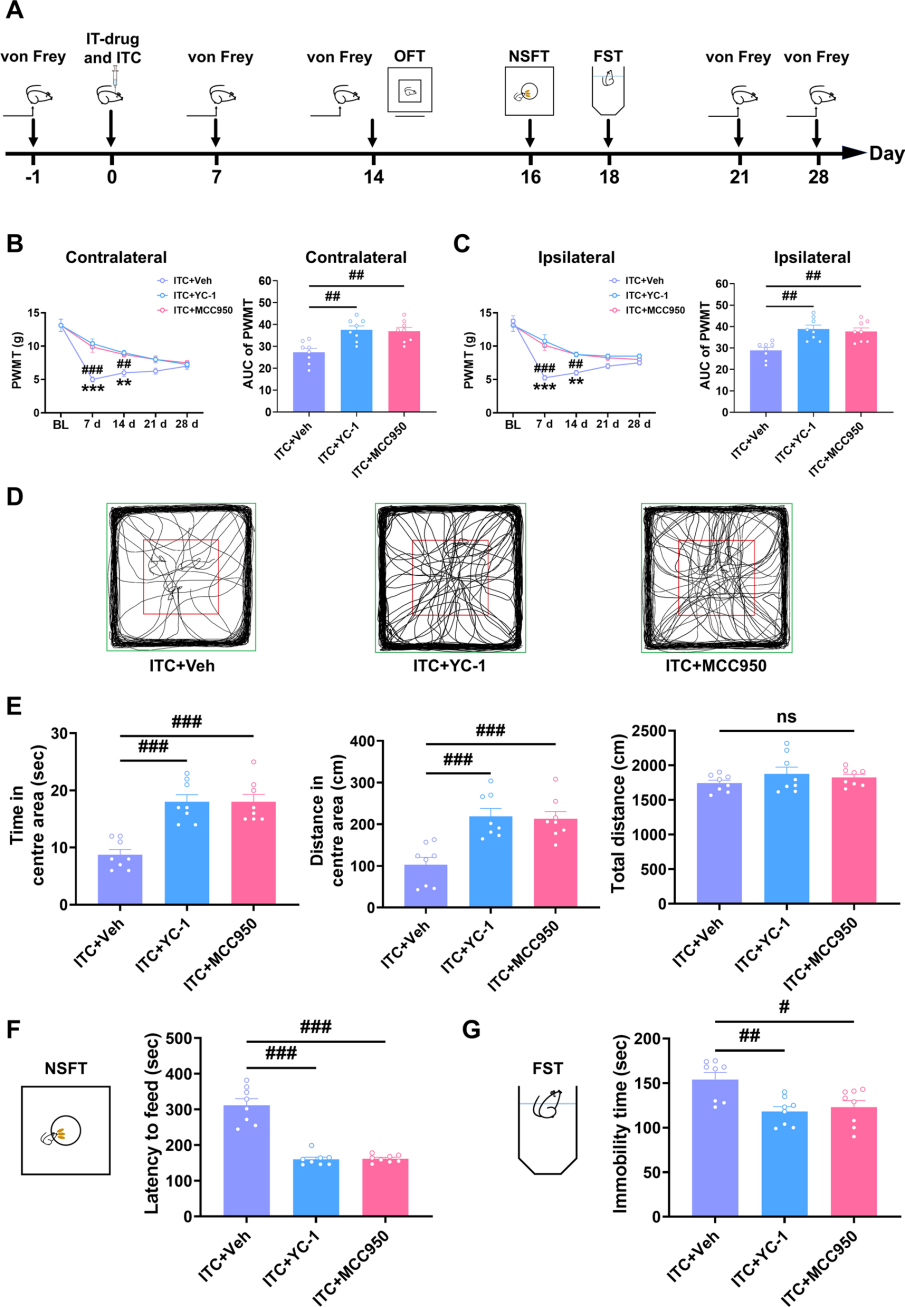
Intra-thalamic injection of YC-1 or MCC950 significantly prevented ITC-induced mechanical allodynia and anxiodepressive-like behaviors in rats. **A** The experimental timeline of surgical procedure and behavior tests. **B** Intra-thalamic injections of YC-1 and MCC950 increased the PWMT of the contralateral hindpaw and the area under the curve of the PWMT following ITC (*n* = 8, PWMT: group, *F*_2, 21_ = 6.30, day, *F*_4, 84_ = 94.81, group × day, *F*_8, 84_ = 7.687, ^###^*p* < 0.001, ITC + YC-1 vs ITC + Veh, **p < 0.001, ITC + Veh vs ITC + MCC950 ; AUC: *F*_2, 21_ = 10.48, ^##^*p* < 0.01). **C** Intra-thalamic injections of YC-1 and MCC950 increased the PWMT of the ipsilateral hindpaw and the area under the curve of the PWMT following ITC (*n* = 8, PWMT: group, *F*_2, 21_ = 6.485, day, *F*_4, 84_ = 81.73, group × day, *F*_8, 84_ = 8.051, ^###^*p* < 0.001, ITC + YC-1 vs ITC + Veh, **p < 0.01, ***p < 0.001, ITC + Veh vs ITC + MCC950; AUC: *F*_2, 21_ = 10.70, ^##^*p* < 0.01). **D** Representative track plot in the OFT. **E** YC-1 and MCC950 increased the time spent and traveled distance traveled in central area, and but had no effect on the total traveled distance in the OFT (*n* = 8, time in central area: *F*_2, 21_ = 21.06, ^###^*p* < 0.001; distance in central area: *F*_2, 21_ = 13.42, ^###^*p* < 0.001; total distance: ns, no significance). **F** YC-1 and MCC950 decreased the latency to feed in the NSFT (*n* = 8, *W*_2, 11.78_ = 31.52, ^###^*p* < 0.001). **G** YC-1 and MCC950 decreased the immobility time in the FST (*n* = 8, *F*_2, 21_ = 7.689, ^#^*p* < 0.05, ^##^*p* < 0.01). Data are expressed as mean ± SEM, one-way ANOVA followed by Tukey test; Welch ANOVA followed by Tamhane’s T2 test

We next inhibited HIF-1α and NLRP3 by intra-thalamic injection of YC-1 (HIF-1α inhibitor) and MCC950 (NLRP3 inhibitor) and assessed mechanical pain sensitivity and anxiety- and depression-like behaviors (Fig. 4A). As shown in Fig. 4B and C, a single intra-thalamic injection of YC-1 and MCC950 prevented the development of bilateral mechanical allodynia compared to the control group, with the analgesic effect lasting for 14 days. In the open field test, intra-thalamic injection of YC-1 and MCC950 in ITC rats significantly increased the time and traveled distance in the central area compared to the control group, but had no effect on the total distance (Fig. 4D and E). In contrast to the control group, intra-thalamic injection of YC-1 and MCC950 remarkably reduced the feeding latency in novelty-suppressed feeding test (Fig. 4F) and the immobility in forced swim test (Fig. 4G). All these results suggest that inhibiting HIF-1α and NLRP3 prevented CPSP-related anxiety and depression.

### Inhibiting HIF-1α and NLRP3 decreased ITC-induced glial activation, neuroinflammation, and oxidative stress in the peri-thalamic lesion site

**Fig. 5:**
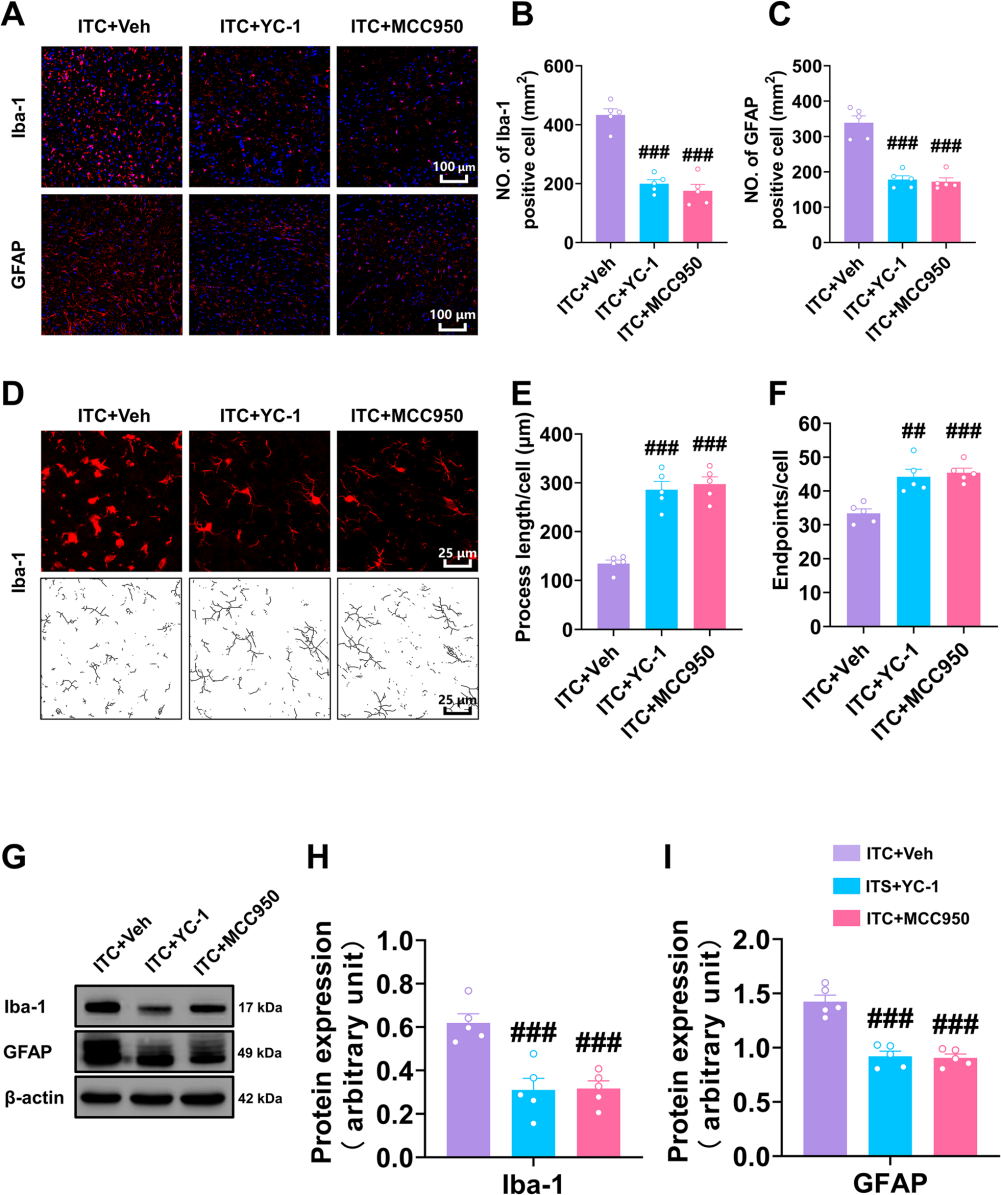
Intra-thalamic injection of YC-1 or MCC950 suppressed ITC-induced activation of microglia and astrocytes in peri-thalamic lesion sites. **A** Representative immunofluorescence images showing the expressions of GFAP and Iba-1 in the peri-thalamic lesion sites. Scale bar = 100 μm. **B** Quantification of cell number showed YC-1 or MCC950 decreased Iba-1 positive cells in the peri-thalamic lesion sites in ITC rats (*n* = 5, *F*_2,12_ = 56.07, ^###^*p* < 0.001 vs ITC + Veh)**. C** Quantification of cell number showed YC-1 or MCC950 decreased GFAP-positive cells in the peri-thalamic lesion sites in ITC rats (*n* = 5, *F*_2,12_ = 44.62, ^###^*p* < 0.001 vs ITC + Veh). **D** Representative magnified images of microglia (top) and the corresponding black-and-white, skeletonized images (bottom) in the peri-thalamic lesion sites. Scale bar = 25 μm. **E** Quantification of process length showed YC-1 or MCC950 increased the process length of microglia in the peri-thalamic lesion sites (*n* = 5, *F*_2,12_ = 44.37, ^###^*p* < 0.001 vs ITC + Veh). **F** Quantification of endpoint showed YC-1 or MCC950 increased the endpoints in microglia in the peri-thalamic lesion sites (*n* = 5, *F*_2,12_ = 22.58, ^##^*p* < 0.01, ^###^*p* < 0.001 vs ITC + Veh). **G** Representative western blots of Iba-1 and GFAP expression in total proteins of the peri-thalamic lesion sites in ITC rats.** H** Quantitative summary result showed YC-1 or MCC950 decreased Iba-1 expression in the peri-thalamic lesion sites in ITC rats (*n* = 5, *F*_2,12_ = 16.17, ^###^*p* < 0.01 vs ITC + Veh). **I** Quantitative summary result showed YC-1 or MCC950 decreased GFAP expression in the peri-thalamic lesion sites in ITC rats (*n* = 5, *F*_2,12_ = 37.29, ^###^*p* < 0.001 vs ITC + Veh). Data are expressed as mean ± SEM, one-way ANOVA followed by the Tukey test

We also examined the effect of inhibiting HIF-1α and NLRP3 on ITC-induced glial activation and neuroinflammatory reaction. As shown in Fig. 5A–C, the number of Iba-1 and GFAP-positive cells in the peri-thalamic lesion site in ITC rats was significantly reduced by intra-thalamic injection of YC-1 and MCC950. The morphological analysis showed that intra-thalamic injection of YC-1 and MCC950 markedly reduced the process length and endpoints in Iba-1-positive cells in the peri-thalamic lesion site of ITC rats (Fig. 5D–F). In line with this immunofluorescent result, the western blotting showed the expression level of Iba-1 and GFAP in the peri-thalamic lesion site of ITC rats were significantly lowered by intra-thalamic injection of YC-1 and MCC950 (Fig. 5G–I). These results suggest blocking HIF-1α and NLRP3 inhibited the hyperactivation of microglia and astrocytes after thalamic hemorrhagic stroke.

**Fig. 6:**
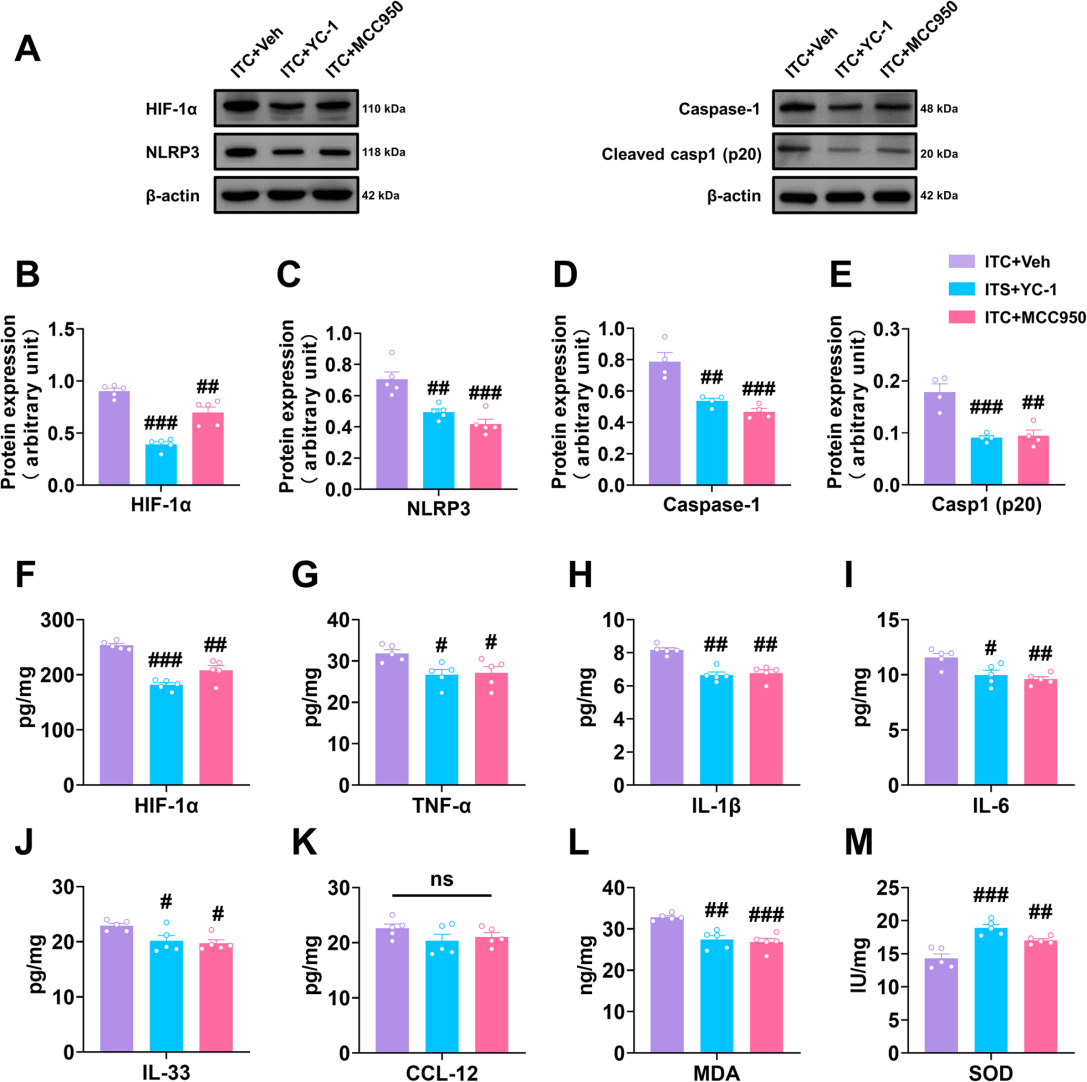
Intra-thalamic injection of YC-1 or MCC950 reduced ITC-induced local inflammation and oxidative stress in the peri-thalamic lesion sites in rats. **A** Representative western blots of HIF-1α, NLRP3, and Caspase-1, and cleaved caspase-1 expression in total proteins of the peri-thalamic lesion sites in ITC rats. **B–E** Quantitative summary result showed intra-thalamic injection of YC-1 or MCC950 reduced HIF-1α (**B**), NLRP3 (**C**), Caspase-1 (**D**), and cleaved caspase-1 (**E**) expression in the peri-thalamic lesion sites in ITC rats (*n* = 5, HIF-1α: *F*_2,12_ = 48.89, ^##^*p* < 0.01, ^###^*p* < 0.001 vs ITC + Veh; NLRP3: *F*_2,12_ = 18.71, ^##^*p* < 0.01, ^###^*p* < 0.001 vs ITC + Veh; *n* = 4, Caspase-1: *F*_2,9_ = 20.56, ^##^*p* < 0.01, ^###^*p* < 0.001 vs ITC + Veh; cleaved caspase-1 p20: *F*_2,9_ = 19.36, ^##^*p* < 0.01, ^###^*p* < 0.001 vs ITC + Veh). **F** ELISA result showed intra-thalamic injection of YC-1 or MCC950 reduced HIF-1α in the peri-thalamic lesion sites in ITC rats (*n* = 5, *F*_2,12_ = 38.82, ^##^*p* < 0.01, ^###^*p* < 0.001 vs ITC + Veh). **G–K** ELISA results showed intra-thalamic injection of YC-1 or MCC950 reduced TNF-α, IL-1β, IL-6 and IL-33 expression, but had no effect on CCL-12 expression in the peri-thalamic lesion sites (*n* = 5, TNF-α: *F*_2,12_ = 5.292, ^#^*p* < 0.05 vs ITC + Veh; IL-1β: *F*_2,12_ = 23.33, ^##^*p* < 0.01 vs ITC + Veh; IL-6: *F*_2,12_ = 9.249, ^#^*p* < 0.05, ^##^*p* < 0.01 vs ITC + Veh; IL-33: *F*_2,12_ = 5.978, ^#^*p* < 0.05 vs ITC + Veh). **L** ELISA result showed intra-thalamic injection of YC-1 or MCC950 reduced the expression of antioxidant factor MDA in the peri-thalamic lesion sites (*n* = 5, *F*_2,12_ = 16.69, ^##^*p* < 0.01, ^###^*p* < 0.001 vs ITC + Veh). **M** ELISA result showed intra-thalamic injection of YC-1 or MCC950 increased the expression of oxidative factor SOD in the peri-thalamic lesion sites (*n* = 5, *F*_2,12_ = 21.45, ^##^*p* < 0.01, ^###^*p* < 0.001 vs ITC + Veh). Data are expressed as mean ± SEM, one-way ANOVA followed by the Tukey test

Next, we investigated the role of HIF-1α and NLRP3 in the inflammatory cascade and oxidative stress after thalamic hemorrhagic stroke by analyzing the expression of several pro-inflammatory cytokines and two key oxidative stress markers, MDA and SOD. Western blotting analysis showed that YC-1 and MCC950 significantly reduced the expression level of HIF-1α, NLRP3, Caspase-1 and cleaved caspase-1 the peri-thalamic lesion site of ITC rats compared to the control group (Fig. 6A–E). ELISA of HIF-1α also indicated that the expression level of HIF-1α in ITC rats was lowered by YC-1 and MCC950 (Fig. 6F). Compared to the control group, the content of pro-inflammatory cytokines TNF-α, IL-1β, IL-6, and IL-33 in the peri-thalamic lesion site of ITC rats were lowered by YC-1 and MCC950, while the expression of chemokine CCL-12 was unaffected (Fig. 6G–K). The oxidative stress indicators in the peri-thalamic lesion site of ITC rats were also measured using ELISA. As shown in Fig. 6L and M, the MDA contents were significantly reduced and SOD activities were significantly increased after intra-thalamic injection of YC-1 and MCC950, implying that blocking HIF-1α and NLRP3 suppressed the oxidative stress induced by thalamic hemorrhagic stroke.

### Post-treatment with repetitive SGB reversed the maintenance of mechanical allodynia and attenuated anxiety and depression in CPSP rats

**Fig. 7:**
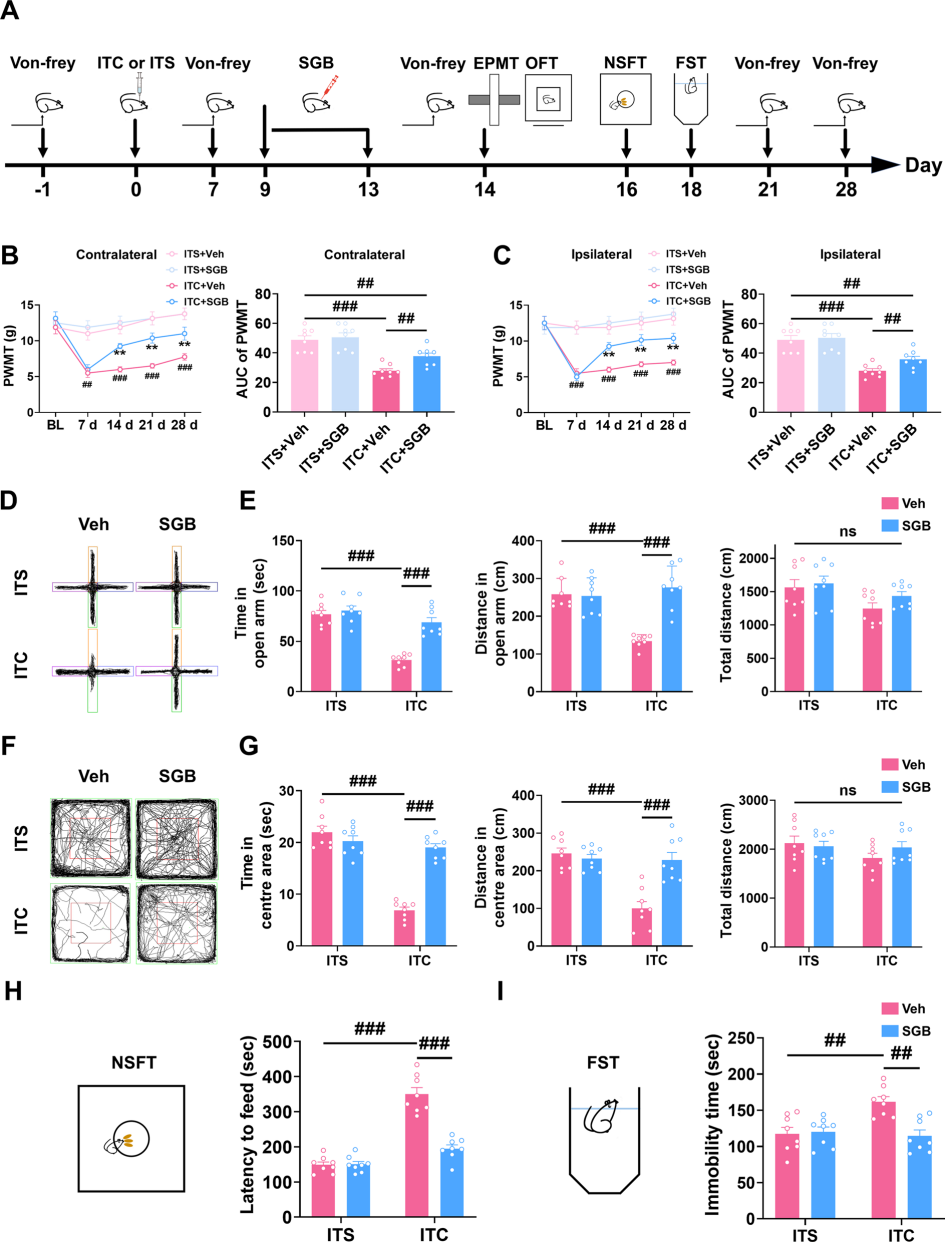
Post-treatment with repetitive SGB reversed the development of mechanical allodynia and anxiodepressive-like behaviors in CPSP rats. **A** The experimental timeline of surgical procedure and behavior tests. **B** Temporal changes of PWMT in contralateral hindpaw after thalamic hemorrhagic stroke and the area under curve of the contralateral hindpaw PWMT (*n* = 8, PWMT: group, *F*_3,28_ = 14.78, day, *F*_4,112_ = 29.22, group × day, *F*_12,112_ = 7.669, **p < 0.01 vs ITC + Veh, ^##^*p* < 0.01, ^###^*p* < 0.001 vs ITS + Veh; AUC: *F*_3,28_ = 18.74, ^##^*p* < 0.01, ^###^*p* < 0.001). **C** Temporal changes of PWMT in ipsilateral hindpaw after thalamic hemorrhagic stroke and the area under curve of the ipsilateral hindpaw PWMT (*n* = 8, PWMT: group, *F*_3,28_ = 15.01, day, *F*_4,122_ = 26.73, group × day, *F*_12,112_ = 9.919, **p < 0.01 vs ITC + Veh, ^###^*p* < 0.001 vs ITS + Veh; AUC: *F*_2,21_ = 19.21, ^##^*p* < 0.01, ^###^*p* < 0.001). **D** Representative track plot in the EPMT. **E** Repetitive SGB increased the time spent and traveled distance in the open arm in ITC group, but had no effect on ITS group (*n* = 8, time in open arm; *F*_3,28_ = 32.45, ^###^*p* < 0.001; distance in open arm; *W*_3.0,13.63_ = 38.47, ^###^*p* < 0.001, total distance: ns, no significance). **F** Representative track plot in the OFT. **G** Repetitive SGB increased the time spent and traveled distance in the central area in ITC group, but had no effect on ITS group (*n* = 8, time in central area: *F*_3,28_ = 58.17, ^###^*p* < 0.001; distance in central *F*_3,28_ = 17.36, ^###^*p* < 0.001; total distance: ns, no significance). **H** Repetitive SGB decreased the latency to feed in the NSFT in ITC group, but had no effect on ITS group (*n* = 8, *F*_3,28_ = 60.17, ^###^*p* < 0.001). **I** Repetitive SGB decreased the immobility time in the FST in ITC group, but had no effect on ITS group (*n* = 8, *F*_3,28_ = 8.058, ^##^*p* < 0.01). Data are expressed as mean ± SEM, one-way or two-way ANOVA followed by Tukey test, Welch ANOVA followed by Tamhane’s T2 test

To evaluate the therapeutic effect of SGB on CPSP, SGB was conducted daily from day 9 to day 13 after thalamic hemorrhagic stroke (Fig. 7A). As shown in Fig. 7B and C, repetitive SGB significantly rescued the bilateral mechanical allodynia in CPSP rats compared to the control group, the analgesic effect persisted throughout 14 to 28 days after thalamic hemorrhagic stroke (Fig. 7B and C). During the whole observational period, repetitive SGB had no effect on the paw withdrawal mechanical threshold in ITS rats (Fig. 7B and C). We also investigated the effect of SGB on CPSP-related anxiety and depression with several behavioral paradigms (Fig. 7A). In the elevated plus maze test, CPSP rats exhibited less time and traveled distance in open arms compared with the ITS group, and these reductions were reversed by repetitive SGB, while in the ITS rats repetitive SGB had no effect (Fig. 7D and E). In accordance with the elevated plus maze test result, CPSP rats exhibited less time and traveled distance in the central area in open field test compared with the ITS group, and these reductions were rescued by repetitive SGB, while in the ITS rats repetitive SGB had no effect (Fig. 7F and G). The total traveled distance in the elevated plus maze test and open field test was unchanged by ITC or repetitive SGB (Fig. 7E and G). In addition, novelty-suppressed feeding test and forced swim test were conducted to assess the anti-depressive effect of SGB. CPSP increased the feeding latency in the novelty-suppressed feeding test and immobility time in the forced swim test compared with the ITS group, these increments were reduced by repetitive SGB, while in the ITS rats repetitive SGB had no significant effect (Fig. 7H and I). These findings imply that post-treatment with repetitive SGB could reverse the mechanical allodynia and anxiety and depression in CPSP rats.

### Repetitive SGB improved cerebral blood flow and suppressed the hyperactivation of microglia and astrocytes in CPSP rats

**Fig. 8:**
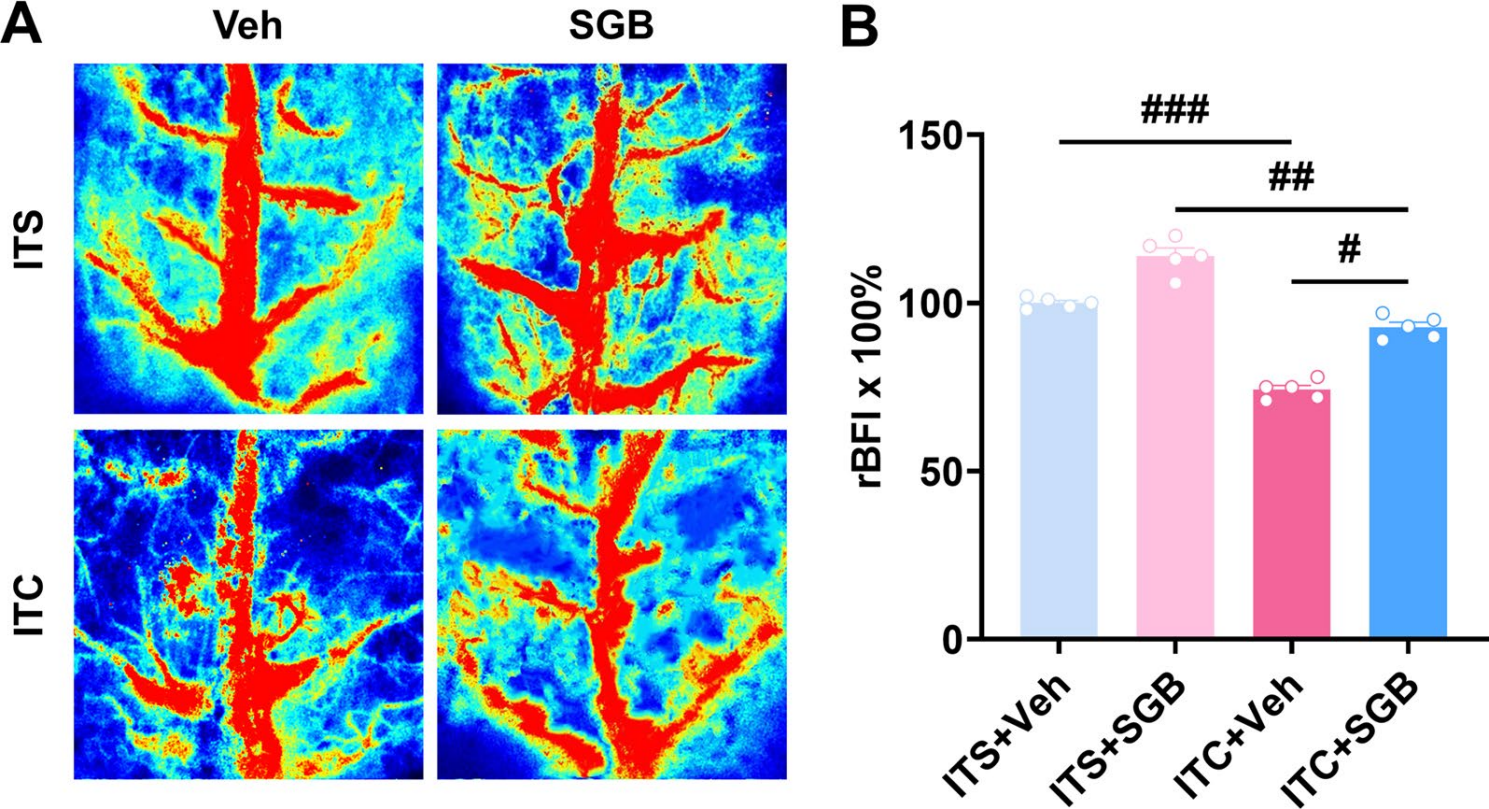
Repetitive SGB increased the cerebral blood flow of CPSP rats. **A** Representative images of cerebral blood flow. **B** Quantitative summary result showed ITC decreased the cerebral blood flow, while repetitive SGB enhanced cerebral blood flow in ITC rats (*n* = 5, F_3, 16_ = 112.2, ^#^*p* < 0.05, ^##^*p* < 0.01, ^###^*p* < 0.001)

Changes in regional cerebral blood flow (rCBF) and insufficient blood and oxygen delivery to the brain may result from a stroke. Thus, on day 14 after thalamic hemorrhagic stroke, we applied LSCI, a well-established technique for monitoring rCBF with high spatial and temporal resolution and real-time imaging, to investigate the effect of SGB on cerebral blood flow in CPSP rats. As shown in Fig. 8A and B, ITC significantly reduced bilateral cortical blood flow compared with ITS group, this reduction was rescued by repetitive SGB in which the cerebral vascular continuity is improved. Moreover, repetitive SGB also remarkably enhanced the cortical blood flow in ITS rats, as seen by the increased visibility of tiny blood vessels (Fig. 8A and B), which is consistent with the sympathetic blocking action of SGB, which may enhance cerebral blood flow under normal condition.

**Fig. 9:**
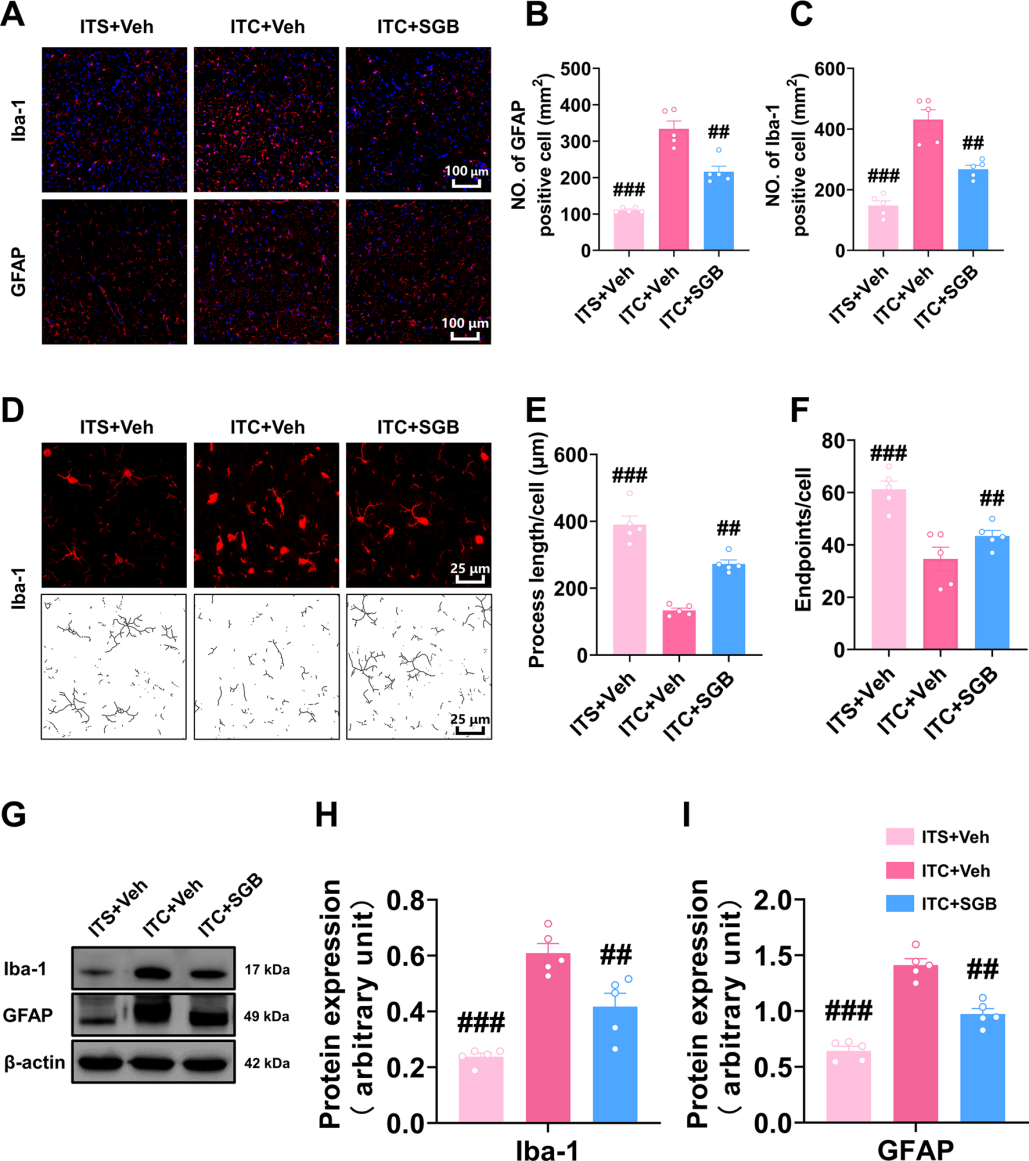
Post-treatment with repetitive SGB inhibited the activation of microglia and astrocytes in peri-thalamic lesion sites of CPSP rats. **A** Representative immunofluorescence images showing the expressions of GFAP and Iba-1 in the peri-thalamic lesion sites. Scale bar = 100 μm. **B, C** Quantification of cell number showed repetitive SGB decreased the total number of GFAP (**B**) and Iba-1 (**C**) positive cells in the peri-thalamic lesion sites of CPSP rats (*n* = 5, Iba-1 *F*_2,12_ = 40.67, ^##^*p* < 0.01, ^###^*p* < 0.001 vs ITC + Veh; GFAP *F*_2,12_ = 99.46, ^##^*p* < 0.01, ^###^*p* < 0.001 vs ITC + Veh). **D** Representative magnified images of microglia (top) and the corresponding black-and-white, skeletonized images (bottom) in the peri-thalamic lesion sites. Scale bar = 25 μm. **E** Quantification of process length showed repetitive SGB increased process length of microglia in the peri-thalamic lesion sites of CPSP rats (*n* = 5, *F*_2,12_ = 59.03, ^##^*p* < 0.01, ^###^*p* < 0.001 vs ITC + Veh). **F** Quantification of endpoint showed repetitive SGB increased endpoint of microglia in the peri-thalamic lesion sites of CPSP rats (*n* = 5, *F*_2,12_ = 15.62, ^##^*p* < 0.01, ^###^*p* < 0.001 vs ITC + Veh). **g** Representative western blots of Iba-1 and GFAP expression in total proteins of the peri-thalamic lesion sites, **H, I** Quantitative summary result showed repetitive SGB decreased Iba-1 (**H**) and GFAP (**I**) expression in the peri-thalamic lesion sites of CPSP rats (*n* = 5, Iba-1: *F*_2,12_ = 28.08, ^##^*p* < 0.01, ^###^*p* < 0.001 vs ITC + Veh; GFAP; *F*_2,12_ = 62.45, ^##^*p* < 0.01, ^###^*p* < 0.001 vs ITC + Veh). Data are expressed as mean ± SEM, one-way ANOVA followed by the Tukey test

We next examine the effect of SGB on glial activation in CPSP rats. As shown in Fig. 9A–C, the number of Iba-1 and GFAP-positive cells in the peri-thalamic lesion site of CPSP rats was increased compared to the ITS group, and this increment was significantly inhibited by repetitive SGB compared to the ITC + Veh group. The morphological analysis of Iba-1-positive cells showed the reduction of process length and endpoints in the CPSP group were significantly rescued by repetitive SGB (Fig. 9D–F). In line with our immunofluorescent results, western blotting showed the enhanced expression level of Iba-1 and GFAP in the ITC group were significantly inhibited by SGB (Fig. 9G–I). These results suggest repetitive SGB was able to suppress the hyperactivation of microglia and astrocytes in the peri-thalamic lesion site of CPSP rats.

### SGB inhibited HIF-1α/NLRP3 signaling as well as its downstream inflammatory response and oxidative stress in CPSP rats

**Fig. 10:**
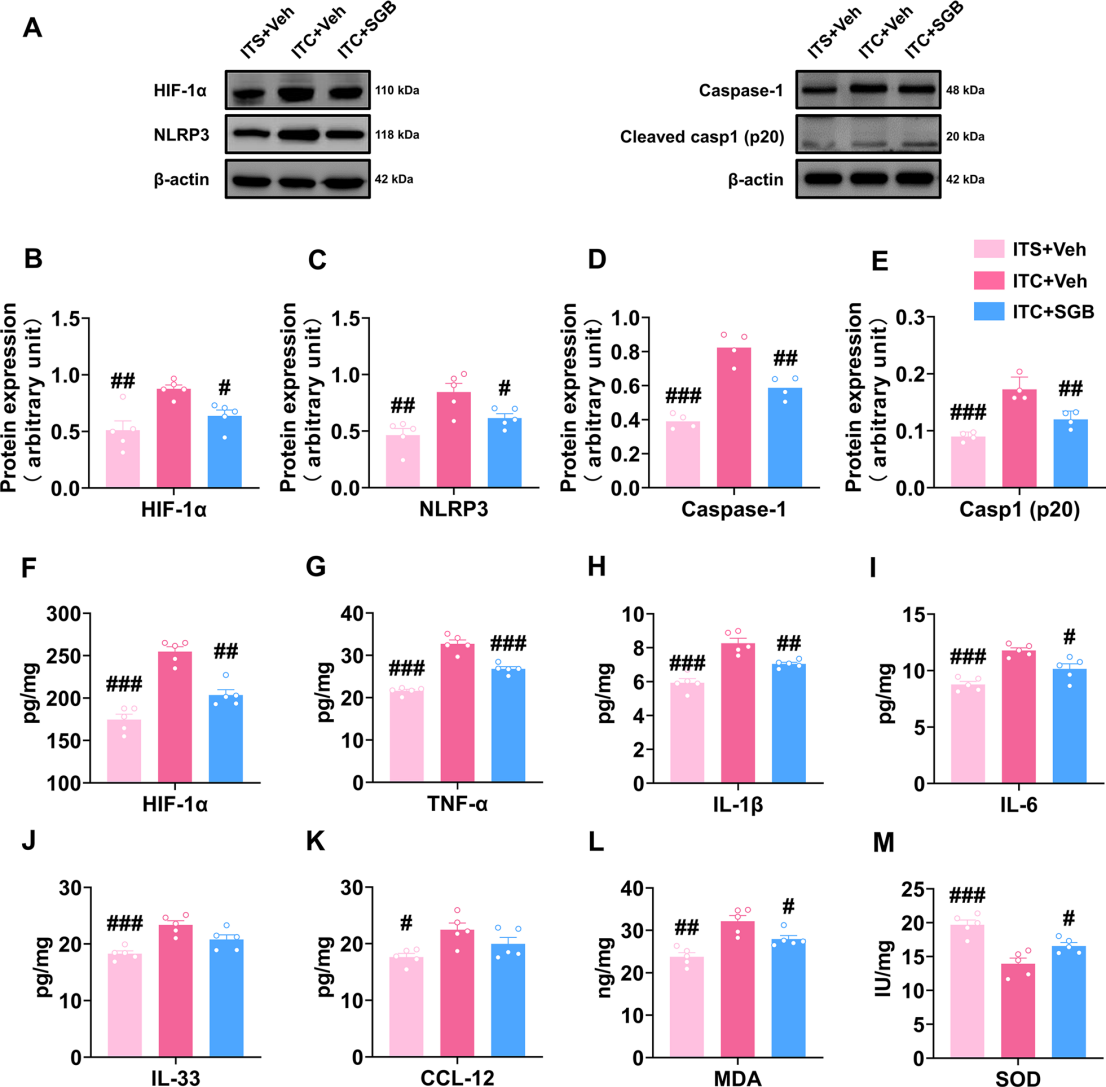
Post-treatment with repetitive SGB reversed local inflammation and oxidative stress in the peri-thalamic lesion sites of CPSP rats. **A** Representative western blotting of HIF-1α, NLRP3, Caspase-1, and cleaved caspase-1 expression in the peri-thalamic lesion sites. **B–E** Quantitative summary result showed repetitive SGB decreased HIF-1α (**B**), NLRP3 (**C**), Caspase-1 (**D**), and cleaved caspase-1 (**E**) expression in the peri-thalamic lesion sites of CPSP rats (*n* = 5, HIF-1α: *F*_2,12_ = 9.896, ^##^*p* < 0.01, ^###^*p* < 0.001 vs ITC + Veh; NLRP3: *F*_2,12_ = 10.40, ^#^*p* < 0.05, ^##^*p* < 0.01 vs ITC + Veh; *n* = 4, Caspase-1: *F*_2,9_ = 38.72, ^##^*p* < 0.01, ^###^*p* < 0.001 vs ITC + Veh; cleaved caspase-1 p20: *F*_2,9_ = 28.21, ^##^*p* < 0.01, ^###^*p* < 0.001 vs ITC + Veh). **F** ELISA result showed repetitive SGB decreased HIF-1α expression in the peri-thalamic lesion sites of CPSP rats (*n* = 5, *F*_2,12_ = 42.10, ^##^*p* < 0.01, ^###^*p* < 0.001 vs ITC + Veh). **G–K** ELISA results showed repetitive SGB decreased TNF-α, IL-1β and IL-6 in the peri-thalamic lesion sites of CPSP rats, but had no effect on IL-33 and CCL-12 (*n* = 5, TNF-α: *F*_2,12_ = 72.48, ^###^*p* < 0.001 vs ITC + Veh; IL-1β: *F*_2,12_ = 26.98, ^##^*p* < 0.01, ^###^*p* < 0.001 vs ITC + Veh; IL-6: *F*_2,12_ = 20.58, ^#^*p* < 0.05, ^#^*p* < 0.05, ^###^*p* < 0.001 vs ITC + Veh; IL-33: *F*_2,12_ = 12.82, ^###^*p* < 0.001 vs ITC + Veh; CCL-12: *F*_2,12_ = 5.570, ^#^*p* < 0.05 vs ITC + Veh). **L** ELISA result showed repetitive SGB decreased the expression of antioxidant factor MDA in the peri-thalamic lesion sites of CPSP rats (*n* = 5, *F*_2,12_ = 15.28, ^#^*p* < 0.05, ^##^*p* < 0.01 vs ITC + Veh). **M** ELISA result showed repetitive SGB increased the expression level of oxidative factor SOD in the peri-thalamic lesion sites of CPSP rats (*n* = 5, *F*_2,12_ = 17.52, ^#^*p* < 0.05, ^###^*p* < 0.001 vs ITC + Veh). Data are expressed as mean ± SEM, one-way ANOVA followed by the Tukey test

As mentioned above, we found that HIF-1α/NLRP3 signaling was critical for the development of CPSP and its related anxiety and depression. Thus, we examined the influence of SGB on HIF-1α/NLRP3 signaling and the downstream inflammatory cascade using western blotting and ELISA. Consistently, the enhanced expression of HIF-1α, NLRP3, Caspase-1, and cleaved caspase-1 in the peri-thalamic lesion sites in CPSP rats was dramatically inhibited by SGB (Fig. 10A–E). ELISA results also showed ITC enhanced HIF-1α expression compared to the ITS group, while SGB rescued this upregulation (Fig. 10F). In contrast to the ITS group, ITC considerably enhanced the expression level of TNF-α, IL-1β, IL-6, IL-33, and CCL-12, whereas SGB significantly reduced the elevation of TNF-α, IL-1β, and IL-6, but had no influence on the expression of IL-33 and CCL-12 (Fig. 10G–K). Additionally, the increased MDA and reduced SOD in the peri-thalamic lesion site of CPSP rats compared to the ITS group were rescued by repetitive SGB (Fig. 10L and M). These findings indicate that SGB exhibited anti-inflammatory and anti-oxidative effects by inhibiting HIF-1α/NLRP3 signaling in the CPSP rats.

### Pharmacological activation of HIF-1α/NLRP3 signaling eliminated the protective effect of SGB on mechanical allodynia and anxiety and depression in CPSP rats

**Fig. 11:**
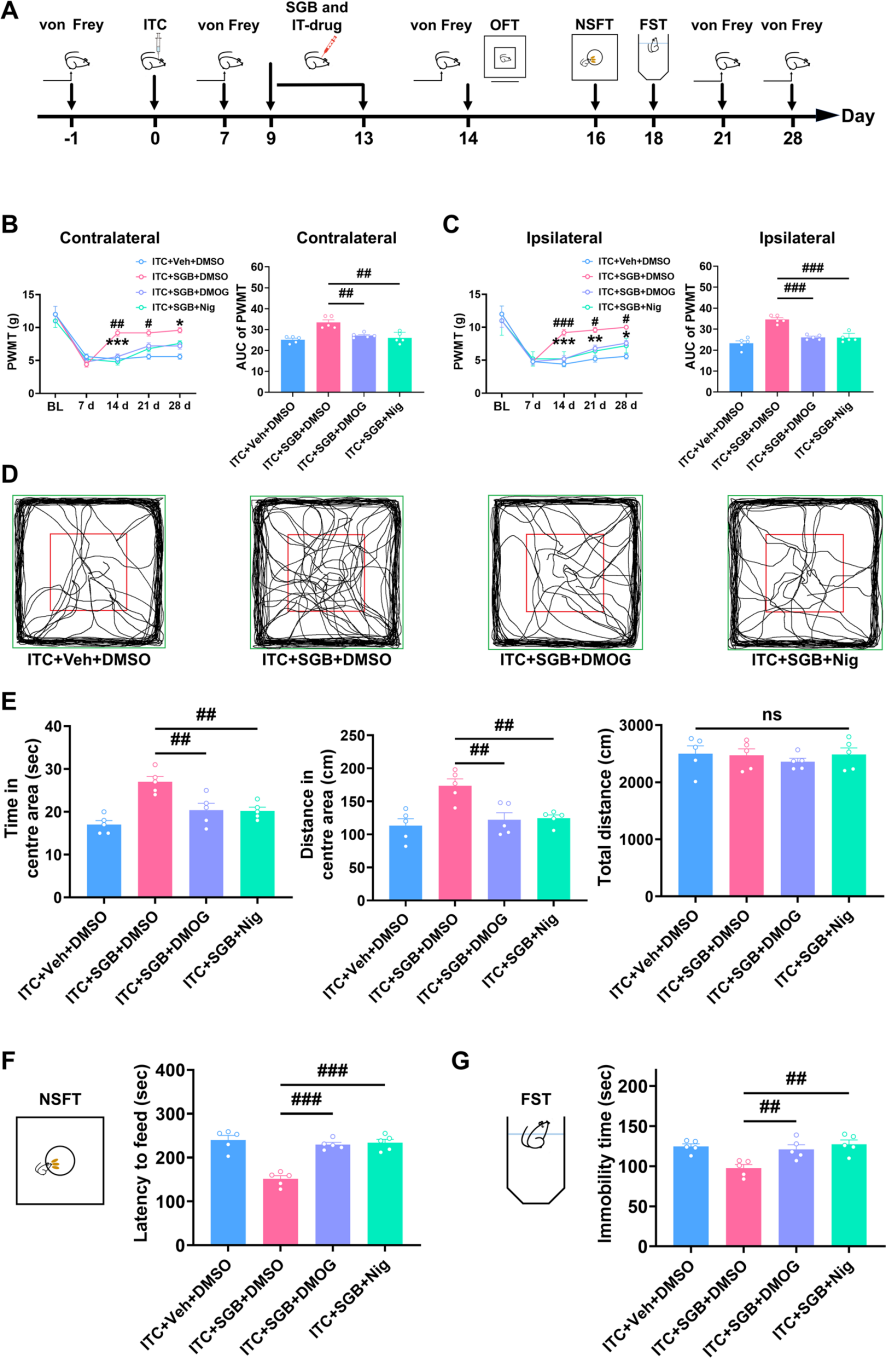
Intra-thalamic injection of DMOG or Nigericin eliminated the therapeutic effects of SGB on mechanical allodynia and anxiety and depression in CPSP rats. **A** The experimental timeline of surgical procedure and behavior tests. **B** Intra-thalamic injections of DMOG or Nigericin decreased the PWMT of the contralateral hindpaw and the area under the curve of the PWMT in ITC rats given SGB (*n* = 5, PWMT: group, *F*_3,16_ = 9.517, day, *F*_4,64_ = 59.90, group × day, *F*_12,64_ = 3.019, ^#^*p* < 0.05, ^##^*p* < 0.01, ITC + SGB + DSMO vs ITC + SGB + DMOG, *p < 0.05, *p* < 0.001, ITC + SGB + DMSO vs ITC + SGB + Nig; AUC: *F*_3,16_ = 14.57, ^##^*p* < 0.01). **C** Intra-thalamic injections of DMOG or Nigericin decreased the PWMT of the ipsilateral hindpaw and the area under the curve of the PWMT in ITC rats given SGB (*n* = 5, PWMT: group, *F*_3,16_ = 17.76, day, *F*_4,64_ = 60.99, group × day, *F*_12,64_ = 2.905, ^#^*p* < 0.05, ^###^*p* < 0.001, ITC + SGB + DMSO vs ITC + SGB + DMOG, *p < 0.05, **p < 0.01,***p < 0.001, ITC + SGB + DMSO vs ITC + SGB + Nig; AUC: *F*_3,16_ = 29.48 ^###^*p* < 0.001). **D** Representative track plot in the OFT. **E** DMOG and Nigericin decreased the time spent and traveled distance traveled in central area, and but had no effect on the total traveled distance in ITC rats given SGB (*n* = 5, time in central area: *F*_3,16_ = 12.60, ^##^*p* < 0.01; distance in central area: *F*_3,16_ = 8.491, ^##^*p* < 0.01; total distance: ns, no significance). **F** DMOG and Nigericin increased the latency to feed in the NSFT in ITC rats given SGB (*n* = 5, *F*_3,16_ = 27.42, ^###^*p* < 0.001). **G** DMOG and Nigericin increased the immobility time in the FST in ITC rats given SGB (*n* = 5, *F*_3,16_ = 8.085, ^##^*p* < 0.01). Data are expressed as mean ± SEM, one-way ANOVA followed by Tukey test

To further confirm that thalamic HIF-1α/NLRP3 signaling mediated the protective effects of SGB on CPSP and comorbid anxiety and depression following thalamic hemorrhage, we pharmacologically activated thalamic HIF-1α and NLRP3 by intra-thalamic injection of nigericin and DMOG at 9, 11, and 13 days in rats given repetitive SGB after intra-thalamic collagenase injection (Fig. 11A). As shown in Fig. 11B and C, compared to the control group, pharmacological activation of thalamic HIF-1α or NLRP3 signaling with nigericin or DMOG in ITC rats significantly eliminated the analgesic effect of repetitive SGB on bilateral mechanical allodynia at 14, 21, and 28 days (Fig. 11B and C). In the open field test, pharmacological activation of thalamic HIF-1α or NLRP3 signaling with nigericin or DMOG significantly reduced the increased time and traveled distance in the central area in ITC rats given repetitive SGB, but had no effect on the total distance (Fig. 11D and E). Additionally, intra-thalamic injection of nigericin or DMOG remarkably increased the feeding latency in novelty-suppressed feeding test (Fig. 11F) and the immobility in forced swim test (Fig. 11G) in ITC rats given repetitive SGB.

### Pre-treatment with repetitive SGB prevented the development of mechanical allodynia and anxiety and depression in CPSP rats

**Fig. 12:**
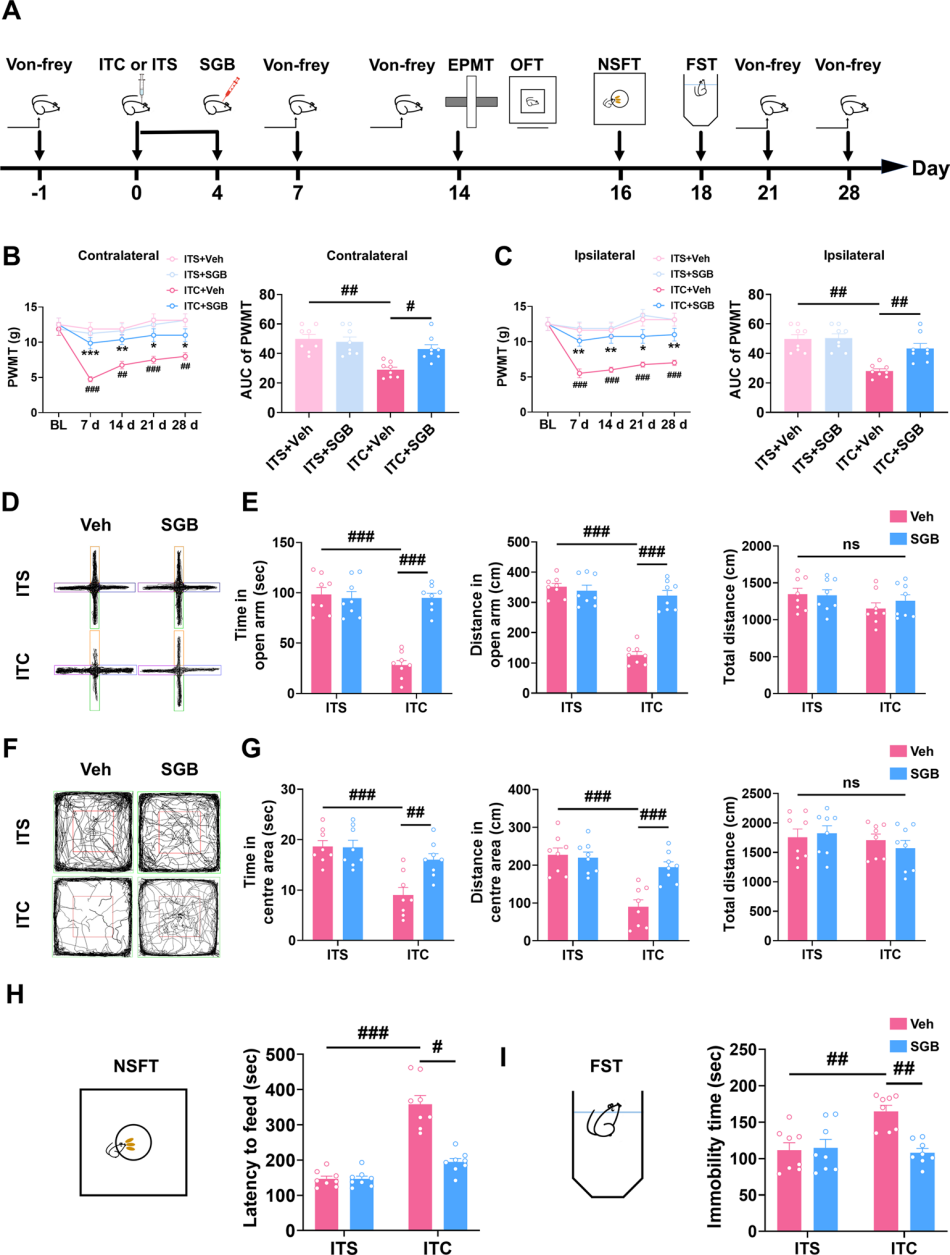
Pretreatment with repetitive SGB prevents the development of mechanical allodynia and anxiodepressive-like behaviors in ITC rats. **A** The experimental timeline of surgical procedure and behavior tests. **B** Temporal changes of PWMT in contralateral hindpaw after thalamic hemorrhagic stroke and the area under curve of the contralateral hindpaw PWMT (*n* = 8, PWMT: group, *F*_3,28_ = 9.268, day, *F*_4,112_ = 10.48, group × day, *F*_12,112_ = 6.246, *p < 0.05, **p < 0.01 vs ITC + Veh, ^###^*p* < 0.001 vs ITS + Veh; AUC: *F*_3,28_ = 18.74, ^#^*p* < 0.05, ^##^*p* < 0.01). **C** Temporal changes of PWMT in ipsilateral hindpaw after thalamic hemorrhagic stroke and the area under curve of the ipsilateral hindpaw PWMT (*n* = 8, PWMT: group, *F*_3,28_ = 10.48, day, *F*_4,122_ = 17.65, group × day, *F*_12,112_ = 7.005, *p < 0.05, **p < 0.01 vs ITC + Veh, ^###^*p* < 0.001 vs ITS + Veh; AUC: *F*_2,21_ = 19.21, ^##^*p* < 0.01). **D** Representative track plot in the EPMT. **E** Pretreatment with repetitive SGB increased the time spent and traveled distance in the open arm in ITC group, but had no effect on ITS group (*n* = 8, time in open arm: *F*_3,28_ = 35.13, ^###^*p* < 0.001; distance in open arm: *F*_3,28_ = 51.39, ^###^*p* < 0.001; total distance: ns, no significance). **F** Representative track plot in the OFT. **G** Pretreatment with repetitive SGB increased the time spent and traveled distance in central area in ITC group, but had no effect on ITS group (*n* = 8, time in central area: *F*_3,28_ = 11.38, ^##^*p* < 0.01, ^###^*p* < 0.001; distance in central area: *F*_3,28_ = 14.80, ^###^*p* < 0.001; total distance: ns, no significance). **H** Pretreatment with repetitive SGB decreased the latency to feed in the NSFT in ITC group, but had no effect on ITS group (*n* = 8, *W*_3.0,15.05_ = 23.77, ^#^*p* < 0.05, ^###^*p* < 0.001). **I** Pretreatment with repetitive SGB decreased the immobility time in the FST in ITC group, but had no effect on ITS group (*n* = 8, *F*_2,21_ = 8.253, ^##^*p* < 0.01). Data are expressed as mean ± SEM, one-way or two-way ANOVA followed by Tukey test, Welch ANOVA followed by Tamhane’s T2 test

Next, we conducted SGB daily from the day of surgery until the fourth postsurgical day to investigate the preventive effect of SGB on CPSP and the related anxiety and depression (Fig. 12A). Compare to the control group, pre-treatment with repetitive SGB significantly prevented the development of bilateral mechanical allodynia in ITC rats, manifested as the increased paw withdrawal mechanical threshold on 7, 14, 21, and 28 days after surgery (Fig. 12B and C). However, pre-treatment with repetitive SGB had no influence on the bilateral paw withdrawal mechanical threshold in ITS group during the whole observation period (Fig. 12B and C). In the elevated plus maze test, repetitive SGB prevented the reduced time and distance traveled in open arms in the ITC group, while SGB had no influence on the ITS group (Fig. 12D and E). Compared to the ITS group, the time and traveled distance in the central area in open field test were decreased in the ITC group, and these reductions were significantly prevented by repetitive SGB, but repetitive SGB had no effect on the ITS group (Fig. 12F and G). The total traveled distance in the elevated plus maze test and open field test was unaffected by ITC or pre-treatment with repetitive SGB (Fig. 12E and G). Consistently, ITC increased the feeding latency of novelty-suppressed feeding test and the immobility time of forced swim test compared with the ITS group, but repetitive SGB prevented these increases (Fig. 12H and I). However, repetitive SGB had no effects on the ITS group (Fig. 12H and I). These findings suggest that pre-treatment with repetitive SGB was sufficient to prevent the development of CPSP and related anxiety and depression.

## Discussion

We found that thalamic hemorrhagic stroke causes mechanical allodynia, anxiety- and depression-like behaviors, and peri-thalamic lesion site HIF-1α/NLRP3 signaling upregulation and glial cell activation. Inhibiting thalamic HIF-1α/NLRP3 signaling prevented thalamic hemorrhage stroke-induced CPSP, anxiety, depression, and peri-thalamic lesion site neuroinflammation. Repetitive SGB post-treatment alleviated CPSP with comorbid anxiety and depression. In CPSP rats, SGB reduced thalamic HIF-1α/NLRP3 signaling upregulation, microglial and astrocytic hyperactivation, inflammatory cytokines, and oxidative stress. Furthermore, repetitive SGB improved cerebral blood flow in CPSP rats. However, pharmacological activation of thalamic HIF-1α/NLRP3 signaling eliminated the therapeutic effect of SGB on CPSP with comorbid anxiety and depression. Finally, repeated SGB also prevented thalamic hemorrhage stroke-induced CPSP with comorbid anxiety and depression. Our study showed that HIF-1α/NLRP3 inflammatory signaling contributed to the CPSP and comorbid anxiety and depression. SGB inhibited HIF-1α/NLRP3 inflammatory signaling and could treat CPSP and comorbid anxiety and depression.

### HIF-1α/NLRP3 inflammatory signaling mediated the development of CPSP and comorbid anxiety and depression

Consistent with our previous studies [14, 34, 35], we confirmed that microinjection of collagenase into unilateral VPL regions of thalamus resulted in long-lasting mechanical pain hypersensitivity, which closely resembles CPSP, a central neuropathic pain caused by thalamic hemorrhagic stroke in patients. It is noteworthy that psychiatric disorders, such as depression and anxiety, are frequently comorbid with neuropathic pain, and depression/anxiety may worsen pain and vice versa [42–45]. In contrast to studies on core pain symptoms, few studies have investigated CPSP’s comorbid emotion in the recent decade. Using a variety of behavior paradigms, we identified anxiety- and depression-like behaviors in CPSP rats, consistent with findings from two very recent studies [15, 46]. Accompanied by the persistent mechanical allodynia, the microglia and astrocytes in the peri-thalamic lesion sites were extremely activated, and HIF-1α and NLRP3 were highly upregulated. Our intra-thalamic siRNA injection assay revealed the functional nature of upregulated HIF-1 and NLRP3, demonstrating that genetic knockdown of thalamic HIF-1 and NLRP3 could prevent mechanical allodynia after thalamic stroke. The pharmacological inhibition of thalamic HIF-1α and NLRP3 with selective inhibitor also confirmed that thalamic HIF-1α/NLRP3 signaling plays a critical role in the development of CPSP. We further found that pharmacological inhibition of thalamic HIF-1α and NLRP3 prevented peri-thalamic lesion site glial activation and inflammatory cytokine upregulation, demonstrating that HIF-1α/NLRP3 signaling causes the inflammatory response after thalamic hemorrhagic stroke. Actually, the HIF-1α/NLRP3 pathway has been recognized as a potential molecular target for treating ischemic stroke and traumatic brain injury because it regulates microglia activation and inflammatory cytokines release in the inflammatory cascade after brain injury [17, 47, 48]. Oxidative stress, a change in the pro-oxidant/antioxidant balance that promotes oxidation, also leads to stroke-related brain injury [49, 50]. Our results showed that inhibiting HIF-1α/NLRP3 signaling resolved the MDA/SOD imbalance after thalamic hemorrhagic stroke, which was corroborated by previous studies showing that HIF-1α and NLRP3 inflammasome activation increases brain oxidative stress after cerebral ischemia in rats [51, 52]. We have demonstrated HIF-1α-primed neuroinflammation promoted CPSP in our earlier work [14]. Consistent with our results, a recent study showed that NLRP3/ASC/caspase-1 dramatically increased following collagenase-induced thalamic pain, while NLRP3 siRNA intra-thalamic microinjection could reduce pain [16]. The most striking finding of this study is that inhibiting thalamic HIF-1α/NLRP3 inflammatory signaling was effective to prevent the anxiety and depression related to CPSP. Current studies of the role thalamus in mood disorders have mostly focused on structural and functional connections, whereas research on its cellular-molecular mechanisms is rare [53–56]. The thalamus, particularly the medial thalamus, is anatomically connected to the anterior cingulate cortex and plays an important role in the expression and experience of emotion, as is well documented [57–60]. In thalamic hemorrhage-induced CPSP, emerging studies have demonstrated that dysregulated local neuroinflammation caused the aberrant excitability and synaptic plasticity in the thalamus and anterior cingulate cortex [9, 11, 13, 14, 61]. Thus, we determined the causal relationship between local neuroinflammation and CPSP-related anxiety and depression by inhibiting thalamic HIF-1α/NLRP3 inflammatory signaling. Our data demonstrated that thalamic HIF-1α/NLRP3 inflammatory signaling was responsible for the comorbid anxiety and depression in CPSP rats. A very recent study confirms our findings that decreasing HIF-1α/NLRP3 in the brain ameliorates lipopolysaccharide-induced depressive-like behavior [20]. The neurocircuit mechanisms through which thalamic HIF-1α/NLRP3 inflammatory signaling causes CPSP-related anxiety and depression are unclear. We hypothesized that neuroinflammatory events could alter neural and synaptic plasticity in the thalamus, which caused functional changes in the output brain regions, such as anterior cingulate cortex and prefrontal cortex, and eventually led to anxiety and depression, since transcranial direct current stimulation of the dorsolateral prefrontal cortex was also shown to improve CPSP and negative mood [62]. Further revealing thalamus-organized neural circuits and molecular mechanisms may be the focus of future research on CPSP with comorbid anxiety and depression, which might pave the way for the precise treatment of CPSP.

### SGB improved CPSP and comorbid anxiety and depression by inhibiting HIF-1α/NLRP3 signaling

SGB, a well-known sympathetic modulation approach, could improve cerebral blood flow and hypoxia and is an effective treatment for several cerebrovascular diseases and pathological pain [26, 27, 63, 64]. Using laser speckle contrast imaging, we have for the first time visualized that SGB improves cerebral blood flow following experimental thalamic hemorrhagic stroke, which were corroborated by other study showing that SGB could alleviate cerebral vasospasm and induce dilation of intracerebral blood vessels in an experimental rat model of subarachnoid hemorrhage [21]. Given the theoretical possibility that it could increase the area of hemorrhage, SGB seemed unsuitable for immediate use in cerebral hemorrhage. However, a previous study has reported that patients with fresh cerebral hemorrhages, immediately upon entrance to the hospital, received SGB without any apparent harm resulting from the block, suggesting that SGB might be an optional and safe therapy for cerebral hemorrhage [65, 66]. Due to the limitations of LSCI technology, we could only monitor changes in blood flow in the superficial cerebral areas. However, a previous study has shown that unilateral SGB was also able to boost oxygenation in deep cerebral regions on the blocked side [67]. Our findings showed that SGB dramatically reduced thalamic HIF-1α overexpression, also indicated that SGB could improve thalamus oxygen supply. Repetitive unilateral SGB restored the increased NLRP3 inflammasome, activated microglia and astrocytes, upregulated pro-inflammatory cytokines, and disrupted MDA and SOD in the peri-thalamic lesion sites, proving that SGB had a significant anti-inflammatory and anti-oxidative role in thalamic stroke. Similar to our findings, Li et al. showed that SGB inhibited Toll-like receptor 4/nuclear factor kappa B signaling pathway and reduced inflammatory response during the ischemic stroke [68]. In addition to suppressing HIF-1α/NLRP3 inflammatory signaling, SGB significantly attenuated thalamic hemorrhage-induced CPSP and comorbid anxiety and depression. However, pharmacological activation of thalamic HIF-1α and NLRP3 significantly eliminated the therapeutic effects of SGB on mechanical allodynia and anxiety- and depression-like behaviors following thalamic hemorrhage, which confirmed that SGB improved CPSP and comorbid anxiety and depression through inhibiting HIF-1α/NLRP3 inflammatory signaling. SGB may have other mechanisms to reduce anxiety and depression. SGB reduced depression-like behaviors in an unexpected chronic moderate stress model owing to an anti-apoptotic mechanism of two stress pathways, the autonomic system and the HPA axis [69]. Anyway, this study broadens our understanding of SGB's action mechanism and informs treatment decisions for CPSP based on increasing the oxygen supply and reducing neuroinflammation. Given the difficulty in translating medications targeting specific molecules in the neuroinflammatory cascade to the clinic, SGB targeting and affecting whole physiological networks is a promising approach for the treatment of CPSP. Actually, previous case studies have shown that SGB offers an effective intervention for CPSP, as the pain subsided rapidly in both intensity and frequency after SGB, and the quality of life was markedly improved [28, 29]. Likewise, SGB was beneficial in the treatment of anxiety symptoms from post-traumatic stress disorder [70–72]. Even though, more clinical studies with larger sample sizes and alternate protocols are needed to further explore the therapeutic potential of SGB for CPSP and related psychiatric disorders.

## Conclusions

In conclusion, our findings demonstrated that a thalamic hemorrhagic stroke resulted in CPSP and comorbid anxiety- and depression-like behaviors. Upregulated HIF-1α/NLRP3 signaling in the peri-thalamic sites activated microglia and astrocytes, releasing pro-inflammatory cytokines and oxidative stress, leading to CPSP and comorbid anxiety and depression. SGB increases cerebral blood flow to suppress HIF-1α/NLRP3 inflammatory signaling, improving the CPSP and comorbid anxiety and depression. Thus, SGB could be used as a promising therapeutic strategy for CPSP and comorbid anxiety and depression symptoms.

## Supplementary Information

**Additional file 1: Figure S1.** Uncropped blots images of Figs. 2, 3, 5, 6, 9 and 10

## Data Availability

The data in this study are available from the corresponding author upon reasonable request.

## Abbreviations

BFI: Blood flow index
CPSP: Central post-stroke pain
DMSO: Dimethyl sulfoxide
ELISA: Enzyme-linked immunosorbent assay
EPMT: Elevated plus maze test
FST: Forced swim test
HIF-1α: Hypoxia-inducible factor 1α
ITC: Intra-thalamic collagenase injection
ITS: Intra-thalamic saline injection
LSCI: Laser speckle contrast imaging
MDA: Malondialdehyde
NLRP3: NLR family pyrin domain containing 3
NSFT: Novelty-suppressed feeding test
OFT: Open field test
PWMT: Paw withdrawal mechanical threshold
rCBF: Regional cerebral blood flow
SGB: Stellate ganglion block
SOD: Superoxide dismutase
VPL: Ventral-posterolateral nucleus
