# Supplementary figures and images for: Stellate ganglion block ameliorated central post-stroke pain with comorbid anxiety and depression through inhibiting HIF-1α/NLRP3 signaling following thalamic hemorrhagic stroke
Source: J Neuroinflammation. 2023 Mar 21;20:82. doi: 10.1186/s12974-023-02765-2 (PMC10031944; doi:10.1186/s12974-023-02765-2)

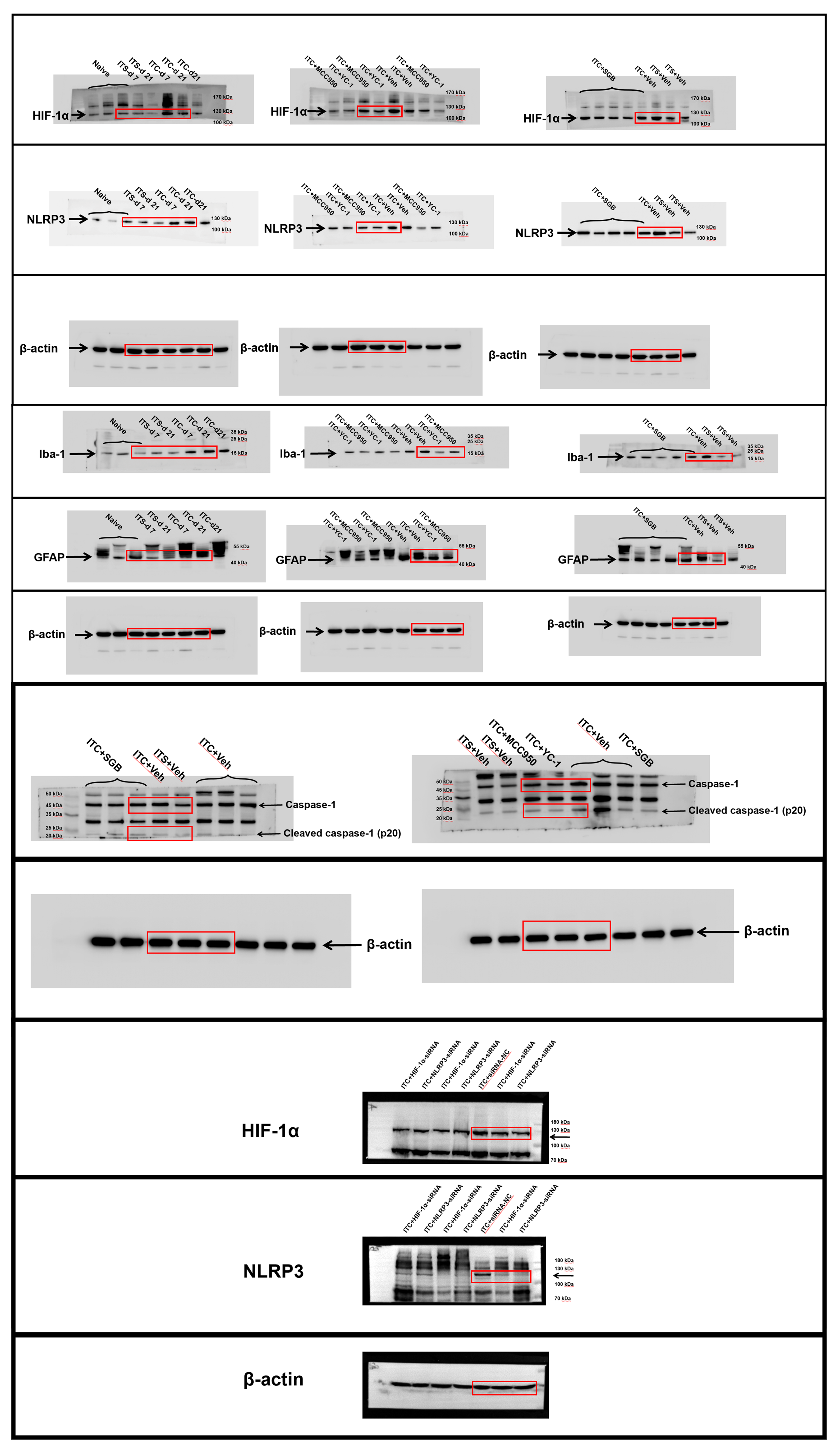

Supplement: Supplementary file 1 — Additional file 1: Figure S1. Uncropped blots images of Figs. 2, 3, 5, 6, 9 and 10 [file 12974_2023_2765_MOESM1_ESM.tif]
